# Individual risk factors associated with SARS-CoV-2 infection during Alpha variant in high-income countries: a systematic review and meta-analysis

**DOI:** 10.3389/fpubh.2024.1367480

**Published:** 2024-07-30

**Authors:** Marta Moniz, Sofia Pereira, Patricia Soares, Pedro Aguiar, Helena Donato, Andreia Leite

**Affiliations:** ^1^NOVA National School of Public Health, Public Health Research Centre, Comprehensive Health Research Center, CHRC, REAL, CCAL, NOVA University Lisbon, Lisbon, Portugal; ^2^Public Health Unit, Amadora Primary Healthcare Cluster, Lisbon, Portugal; ^3^Department of Epidemiology, Instituto Nacional de Saúde Doutor Ricardo Jorge (INSA), Lisbon, Portugal; ^4^Documentation and Scientific Information Service, Centro Hospitalar e Universitário de Coimbra, Coimbra, Portugal

**Keywords:** SARS-CoV-2, infectious disease, risk-factors, systematic review, meta-analysis

## Abstract

**Objectives:**

This study aimed to systematically appraise risk factors associated with SARS-CoV-2 infection in high-income countries during the period of predominance of the Alpha variant (January 2020 to April 2021).

**Methods:**

Four electronic databases were used to search observational studies. Literature search, study screening, data extraction and quality assessment were conducted by two authors independently. Meta-analyses were conducted for each risk factor, when appropriate.

**Results:**

From 12,094 studies, 27 were included. The larger sample size was 17,288,532 participants, more women were included, and the age range was 18–117 years old. Meta-analyses identified men [Odds Ratio (OR): 1.23, 95% Confidence Interval (CI): 1.97–1.42], non-white ethnicity (OR: 1.63, 95% CI: 1.39–1.91), household number (OR: 1.08, 95% CI: 1.06–1.10), diabetes (OR: 1.22, 95% CI: 1.08–1.37), cancer (OR: 0.82, 95% CI: 0.68–0.98), cardiovascular diseases (OR: 0.92, 95% CI: 0.84–1.00), asthma (OR: 0.83, 95% CI: 0.75–0.92) and ischemic heart disease (OR: 0.82, 95% CI: 0.74–0.91) as associated with SARS-CoV-2 infection.

**Conclusion:**

This study indicated several risk factors for SARS-CoV-2 infection. Due to the heterogeneity of the studies included, more studies are needed to understand the factors that increase the risk for SARS-CoV-2 infection.

**Systematic review registration:**

https://www.crd.york.ac.uk/prospero/display_record.php?ID=CRD42021244148, PROSPERO registration number, CRD42021244148.

## Introduction

In December 2019, an atypical pneumonia outbreak was registered in the Wuhan province. The Chinese authorities later identified a new virus – severe acute respiratory syndrome coronavirus 2 (SARS-CoV-2) – as the pathogen originating the outbreak. The globalized world propelled its dissemination, and in just a few months, COVID-19 reached several countries. On March 11th of 2020, the World Health Organization declared COVID-19 as a pandemic (1), which on August 24th of 2022 had already infected almost 600 million people and caused over 6 million deaths globally (2). Due to its utmost impact on overall human life, the United Nations Organization has declared COVID-19 a social, human, and economic crisis (3).

Recognizing the rapid spread and severe impact of the pandemic, researchers have been working to understand the virus and its effects. Hence, a large volume of literature on SARS-CoV-2 infection has been published, namely epidemiological characteristics of positive cases and outcomes. Thus, leading to the development of systematic review (SR) on risk factors for developing infection, severe disease and mortality. SRs identified several factors associated with severe COVID-19, such as being older (4, 5), male (4, 5), having a high body mass index (4, 5) and multiple previous comorbidities [e.g., hypertension, diabetes, cardiovascular disease, chronic obstructive pulmonary disease (4), and active cancer (5)]. Other SRs identified factors associated with SARS-CoV-2 infection, such as lack of protective personal equipment, being female, poor access to healthcare, high volume of tourism and high population density. However, SRs on risk factors for infection were mainly restricted to specific subgroups (i.e., health workers) (6) or continents (i.e., Africa) (7), in which factors might be different to other contexts due to specific contacts and demographics.

Furthermore, when comparing infection rates, a disparity seems to emerge between different economic contexts. High-income countries reported higher infection rates than low-income countries (8), which might be partially explained by different contextual factors, medical infrastructures, and human and technical resources. There was only one SR in high-income countries, conducted in the UK, regarding risk factors for SARS-CoV-2 infection. This SR found that older adults, being male, black, having previous comorbidities, living in urban areas and more deprived areas were associated with a higher risk of SARS-CoV-2 infection. However, this search ended in early pandemic stages (April 2020) and was restricted to England and Wales (9).

Additionally, the evolving understanding of risk factors revealed some unique characteristics of COVID-19. Although COVID-19 is a respiratory manifestation, evidence shows that some risk factors for SARS-CoV-2 infection are different from other infectious respiratory diseases in high-income countries, i.e., pneumonia was more common in women and COVID-19 was more common in men (10). There is also contradictory evidence regarding the effect of some diseases, such as diabetes (11, 12) and cancer (13, 14). Moreover, SARS-CoV-2 variants show different transmissibility between them depending on both characteristics of the variants and the population where it spreads, translating into different case severity (15, 16). The Delta variant already seemed more transmissible than Alpha, i.e., showing differences in the characteristics of index cases (17). In the majority of high-income countries, the Alpha variant was the most predominant variant responsible for SARS-CoV-2 epidemic surges between the end of 2020 and the first half of 2021 (17, 18). Given these complexities and heterogeneity, there is a need for focused research on specific periods and contexts. Thus, given the contextual differences between high- and low-income countries and possible differences in the risk of infection according to different SARS-CoV-2 variants, we aim to systematically appraise and quantify the risk factors associated with SARS-CoV-2 infection during the period of predominance of the Alpha variant in high-income countries.

## Methods

This SR protocol has been developed according to the Preferred Reporting Items for Systematic review and Meta-Analysis Protocols (PRISMA-P) (19) and reported in accordance with MOOSE (Meta-analysis Of Observational Studies in Epidemiology) guidelines (Supplementary File 1) (20). We have registered the protocol in the International Prospective Register of Systematic Reviews (PROSPERO), registration ID number: CRD42021244148.

### Data sources and search strategy

The data sources comprised PubMed; Web of Science; EMBASE; MedRxiv, and international conferences (European Scientific Conference on Applied Infectious Disease Epidemiology, ESCAIDE) relevant for this matter, from 2020 and 2021. The World Congress on Public Health (WCPH) and the European Public Health Conference (EPHC) were also considered, but the abstracts presented in these two conferences were published, therefore appearing in the searched databases. The databases were searched from 1/1/2020 to 22/4/2021 when the Alpha variant was predominant in the majority of high-income countries (18) and the last search was conducted on 31/5/2021.

Search terms (text words and Mesh terms) were drawn up for three search concepts: SARS-CoV-2, risk factors, and high-income country. The search in the conference abstract book was done using the words “COVID-19” or “SARS-CoV-2.” High-income countries were defined according to the classification from the World Bank (21). The detailed search strategy is provided in Supplementary File 2. The literature search was performed by two authors, an investigator and a librarian (M.M. and H.D., respectively).

### Inclusion and exclusion criteria

**Figure 1:**
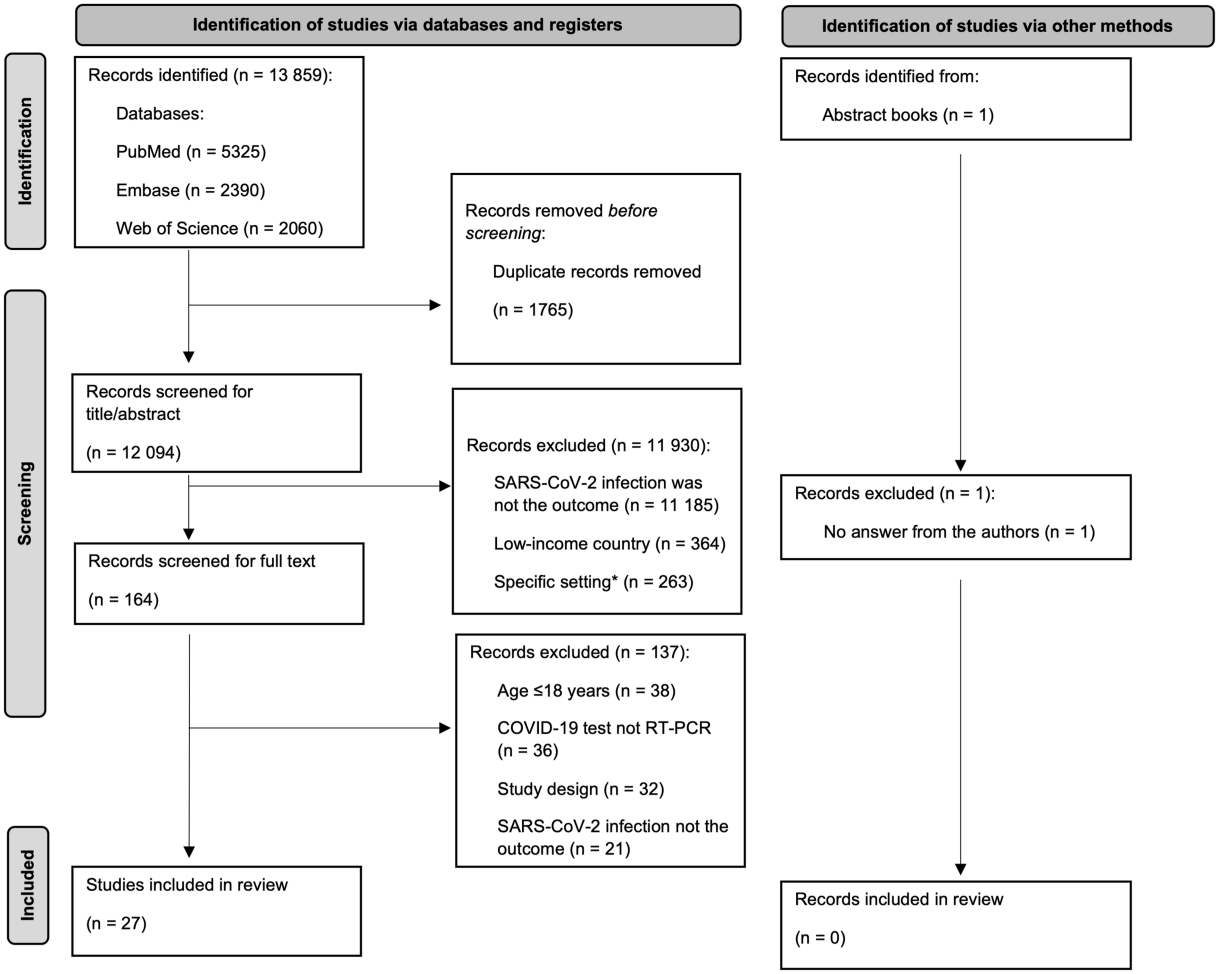
Diagram of study selection, adapted from PRISMA group 2020 flow diagram (high-income countries, 2020–2021).

We included all Portuguese, English, French, Spanish and Italian studies that evaluated the risk factors for SARS-CoV-2 infection in high-income countries with a confirmed Polymerase chain reaction (PCR) SARS-CoV-2 positive test result on people 18 years old or more. After polling the articles and eliminating duplicates, a manual review of titles and abstracts was performed, screening for relevant topics and keywords. Similar studies, in title and authors, found in different databases were screened and confirmed to have different objectives. We excluded articles covering reinfection, specific settings and populations (e.g., health workers, schools, hospitalized patients, pregnant women and people with disabilities). We also excluded articles where diagnosis of SARS-CoV-2 infection was self-reported, or the case definition was a composite of various tests (PCR, antigen, blood samples), and suspected and/or clinically diagnosed cases. Genetic factors were also excluded due to the complexity of the analysis. Environmental factors were excluded due to their specific time–space patterns, thus challenging pooled estimates. Articles lacking information on SARS-CoV-2 infection measurement or population age were excluded for consistency. See Figure 1 for a detailed summary of the selection process.

All individual-based study designs were potentially eligible; however, we have decided to exclude Letters to Editors, Editorials, Comments, Opinions and Ecological studies to analyze more robust information, considering the growing volume of publications. The eligibility criteria were applied by two authors independently (M.M. and S.C.) to titles/abstracts for full-text assessment. References management and screening was carried out using the Rayyan website (22).

### Data extraction and quality assessment

We used a random sample (10%) to pilot the extraction form and two authors (M.M and S.C.) extracted the data. After testing, we adjusted the extraction form including more fields to characterize the studies, namely sample description and controlling factors. Thus, data was extracted using a standardized form which included first author, title, year of publication, study design, study location and duration, study population, data source, sample size, sample description (age and sex), factors identified, estimated measure of effect, and control factors for the statistical analysis. Sample description was deemed important for comparison between studies.

Quality assessment of the studies was conducted by the same two authors using the Joanna Briggs Institute tool for analytical cross-sectional studies (23) and Newcastle-Ottawa Scale (NOS) for case–control and cohort studies (24). Since there is no universal criterion for high-quality studies, we considered those scoring ≥7 as high-quality, a cut-off commonly used in the literature (25). Conflicts between raters in classifying individual items of the abovementioned tools were resolved by discussion with a third author (A.L.).

### Data analysis

For each study, we undertook a descriptive characterization. When at least two studies reported an exposure in a consistent way (same reference and categories), these were combined in a meta-analysis (26, 27). To obtain pooled estimates of SARS-CoV-2 infection risk factors while improving results comparability, we chose to include a single effect measure, decided as the most often reported. In studies that reported several multivariable-adjusted effect estimates, we selected the one that adjusted for more potential confounders (26–28). Each study was weighted in the meta-analysis using the inverse variance of the effect estimate (29).

Heterogeneity between estimates was assessed using the *I*^2^ statistic, with higher values reflecting increased heterogeneity. For higher heterogeneity coefficients (*I*^2^) with statistically significant tests rejecting homogeneity, we used a random-effects model; otherwise, we chose a fixed-effects model (30). We performed sensitivity analyses for ethnicity, diabetes and comorbidities, giving greater depth to the analysis, since they contained various categories within, susceptibly for SARS-CoV-2 infection might be misled. We performed two sensitivity analyses for ethnicity: without the category “other” and dividing the variable into the three ethnicities described (Black, Hispanic and Asian). Comorbidities were arranged into groups of diseases: respiratory diseases (asthma, COPD – chronic obstructive pulmonary disease, respiratory diseases), cardiovascular diseases (arrhythmia, heart failure, cardiomyopathy, ischemic heart disease, cardiovascular diseases), neurological diseases (Alzheimer, degenerative diseases, Parkinson, Parkinsonism and movement disorders, dementia, stroke and transient cerebral ischemia, cerebral hemorrhage) and autoimmune diseases (rheumatoid arthritis, autoimmune diseases). For diabetes we performed an analysis with the studies that analyzed the same type of diabetes. Publication bias was assessed by visually inspecting funnel plots. Statistical analyses were conducted in SPSS version 28.0.1.0 (31).

## Results

The search strategy identified 13,859 records. After removing the duplicates, 12,094 records were screened for title and abstract and full-text screening was performed in the 164 remaining records, from which 27 met the inclusion criteria and were included in this systematic review. An abstract from the ESCAIDE 2021 workbook was identified but excluded, as we tried to obtain more information from the authors but did not get any answer (Figure 1).

From the 27 studies, most studies were cohort (48.1%) (10, 32–43), followed by case–control (29.6%) (44–51) and cross-sectional (22.2%) (52–57) studies. Most studies were set in Europe (44.4%) (10, 32–34, 36, 37, 41–43, 46, 47, 50), followed by North America (29.6%) (35, 38, 39, 45, 49, 53–56) and Asia (25.9%) (40, 44, 48, 51, 52, 57). Sample size was heterogenous, ranging from 310 (43) to 17,288,532 (37) individuals. Participants age varied widely (range: 18–117 years old), with the majority of participants being mostly females (70.4%) (10, 32–35, 39–42, 45, 46, 48–57). The odds-ratio (OR) was the effect measure most often used (81.5%) (32, 34, 36, 37, 39–57), followed by risk ratio (7.4%) (10, 34), hazard ratio (3.7%) (33) and median difference (3.7%) (35). One study did not report a measure of effect, and only reported the *p*-value from the *χ*^2^ test and Fisher’s exact test to evaluate the differences between positive and negative cases of SARS-CoV-2 infection (38).

Education was the only factor that demonstrated consistent results in the studies in which it was reported, with lower levels of education indicating a greater risk of SARS-CoV-2 infection (34, 39, 42, 50). Income was measured both with Social Deprivation Index (TSDI) (10, 34, 42, 45) or household income (50, 56) and both approaches found that a lower economic level was associated with a higher risk of infection. Two studies reported alcohol drinking history, one did not show any association with infection risk (45) and the other found an association with negative test (50). Smoking history was reported in seven studies, being one of them related to the influence of early factors in the risk of infection, demonstrating that maternal smoking around birth was associated with a higher risk of SARS-CoV-2 infection (32). Smoking history was associated with a higher risk of infection in two studies (10, 38) and two others reported a negative association (44, 53). The remaining studies did not show any association (46, 50).

Other risk factors were more frequently reported, such as sex (55.5%), ethnicity (44.4%), age (40.7%), economic conditions (25.9%), household conditions (14.8%) and comorbidities (51.9%), with cancer/malignancy and hypertension being the most prevalent.

Despite being reported more than once, for some risk factors [smoking status (10, 33, 34, 38, 44, 46, 53), education (34, 39, 42, 50), alcohol drinking status (34, 40, 45, 50), and economic conditions (10, 34, 42, 45, 50, 56)], it was not possible to conduct a meta-analysis or include all the studies in a single meta-analysis due to different variable categories or different variable types (continuous/categorical), and/or different effect measures.

For insurance (52, 54) and age (39, 42, 46, 49, 50, 53), only a fraction of the studies was combined due to different classifications. Age was reported as continuous or categorical, and for the meta-analysis, we extracted the measure of effect for continuous measurement since it was the type most often reported. From the studies that reported age as a continuous variable, one was not included since the confidence interval was not available, thus we lacked information to perform the meta-analysis (53).

Sex was the only variable reported consistently among all the studies identified, thus, the meta-analysis for sex included all the studies reporting sex. Table 1 summarizes the key characteristics of the studies included, while more detailed information is available in Supplementary File 3.

**Table 1:** Summary of the included studies considering risk factors for SARS-CoV-2 infection, *N* = 27 (high-income countries, 2020–2021).

First author	Study design	Location	Duration	Data source	Sample size	Factors identified	Quality assessment
Altug Didikoglu, 2021 (32)	Cohort	England, United Kingdom	16 March 2020–21 December 2020	Database – UK Biobank	43,428	Early life factors	5/8
Angel Vila-Córcoles, 2020 (33)	Cohort	Tarragona, Spain	1 March 2020–23 May 2020	Database – CAPAMIS Research	79,083	Underlying comorbidities or using chronic medications	8/8
Ariel Israel, 2020 (44)	Case–control	Israel	Beginning of the disease outbreak – 16 May 2020	Electronic health records – Clalit Health Services	24,906	Smoking habits	8/9
Bing Zhang, 2021 (45)	Case–control	California, United States of America	1 March 2020–10 June 2020	Electronic health records – University of California Health system	861	Use of chronic acid suppressors	7/9
Claire L. Niedzwiedz, 2020 (34)	Cohort	England, United Kingdom	16 March 2020–3 May 2020	Database – UK Biobank	392,116	Ethnicity and socioeconomic position	7/8
Ehab Hamed, 2020 (57)	Cross-sectional	Qatar	10 February 2020–30 April 2020	Electronic health records – publicly funded primary health care settings in the state of Qatar	962	Diagnosis of hematological abnormalities	6/8
Eyrun F. Kjetland, 2020 (46)	Case–control	Norway	1 January 2020–6 April 2020	Electronic records – Oslo University Hospital; Online survey	116,678	Demographic, social, health risk and environmental factors	5/9
Farhaan S. Vahidy, 2020 (56)	Cross-sectional	Houston, United States of America	5 March 2020–31 May 2020	Electronic health records – Houston Methodist	20,228	Ethnicity and race	5/8
Farhaan S. Vahidy, 2021 (55)	Cross-sectional	Houston, United States of America	6 March 2020–22 August 2020	Electronic health records – Houston Methodist	96,473	Sex	6/8
Frederick K Ho, 2020 (10)	Cohort	England, United Kingdom	16 March 2020–3 May 2020	Database – UK Biobank	1,525	Demographic, lifestyle, socioeconomic and clinical risk factors	7/8
Giuseppe Mancia, 2020 (47)	Case–control	Lombardy, Italy	21 February 2020–11 March 2020	Databases – Lombardy Regional Health Service	37,031	Use of angiotensin-receptor blockers (ARBs) and angiotensin-converting–enzyme (ACE) inhibitors	7/9
Harmony R. Reynolds, 2020 (35)	Cohort	New York, United States of America	1 March 2020–15 April 2020	Electronic health records – New York University (NYU) Langone Health	12,594	Use of renin-angiotensin-aldosterone system inhibitors	7/8
Jeongkuk Seo, 2020 (58)	Case–control	South Korea	Beginning of the disease outbreak – 15 May 2020	Database – South Korea Health Insurance Review and Assessment Service	4,932	Use of renin-angiotensin-aldosterone system inhibitors	7/9
Jose L. Pablos, 2020 (36)	Cohort	Spain	7 April 2020–17 April 2020	Database – public Research network for the Investigation of Inflammation and Rheumatic Diseases (RIER)	29,931	Diagnosis of chronic inflammatory and autoimmune rheumatic disease	5/8
Kuan-Han H. Wu, 2021 (49)	Case–control	Michigan, United States of America	1 March 2020–29 July 2020	Michigan Medicine biorepository; Online survey	8,041	Demographic, lifestyle, socioeconomic and clinical risk factors	5/9
L. Silvia Muñoz-Price, 2020 (54)	Cross-sectional	Milwaukee, United States of America	12 March 2020–31 March 2020	Electronic health records – Froedtert and the Medical College of Wisconsin	2,595	Race	8/8
Leonard E Egede, 2020 (53)	Cross-sectional	Wisconsin, United States of America	1 March 2020–10 July 2020	Electronic health records – Froedtert and the Medical College of Wisconsin	31,549	Ethnicity and race	8/8
Marc Chadeau-Hyam, 2020 (50)	Case–control	England, United Kingdom	16 March 2020–18 May 2020	UK Biobank	4,509	Demographic, social, health risk, medical and environmental factors	6/9
Rohini Mathur, 2021 (37)	Cohort	England, United Kingdom	1 February 2020–3 August 2020; 1 September 2020–31 December 2020	Database – OpenSAFELY platform	17,288,532	Ethnicity	7/8
Sachin J Shah, 2020 (38)	Cohort	San Francisco, United States of America	3 February 2020–31 March 2020	Electronic health records – University of California, San Francisco	316	Demographic and medical and factors	5/8
Sara J. Cromer, 2020 (39)	Cohort	New England, United States of America	1 February 2020–21 June 2020	Electronic health records – Mass General Brigham	57,865	Demographic risk factors	8/8
Seon Cheol Park, 2021 (52)	Cross-sectional	South Korea	3 January 2020–31 May 2020	Database – South Korea Health Insurance Review and Assessment Service	219,729	Underlying comorbidities	6/8
Seung Won Lee, 2020 (40)	Cohort	South Korea	1 January 2020–15 May 2020	Database – South Korea Health Insurance Review and Assessment Service	216,418	Diagnosis of mental illness	7/8
Wonjun Ji, 2020 (51)	Case–control	South Korea	Beginning of the disease outbreak – 15 May 2020	Database – South Korea Health Insurance Review and Assessment Service	219,961	Underlying comorbidities	7/9
Xiude Fan, 2021 (41)	Cohort	United Kingdom	16 March 2020–29 June 2020	Database – UK Biobank	9,469	Use of acid- suppressants	7/8
Yizhou Yu, 2021 (42)	Cohort	United Kingdom	16 March 2020–26 July 2020	Database – UK Biobank	13,338	Diagnosis of dementia, Alzheimer disease or Parkinson disease	7/8
Zahra Raisi-Estabragh, 2021 (43)	Cohort	England, United Kingdom	16 March 2020–22 August 2020	Database – UK Biobank	310	Baseline cardiovascular magnetic resonance (CMR) phenotypes	5/8

### Quality assessment

Most cohort studies were rated 7 or 8 out of 8 points, mainly lacking representativeness of the exposed cohort or comparability at the baseline; most case–control studies were rated 8 out of 9 points lacking mostly representativeness of cases or with different methods of ascertainment of exposure for cases and controls; and cross-sectional studies were rated 6 out of 8 with the most common gaps being related to the identification and analysis of confounding factors. The full quality assessment of all included studies is in Supplementary File 4.

### Meta-analysis

**Figure 2:**
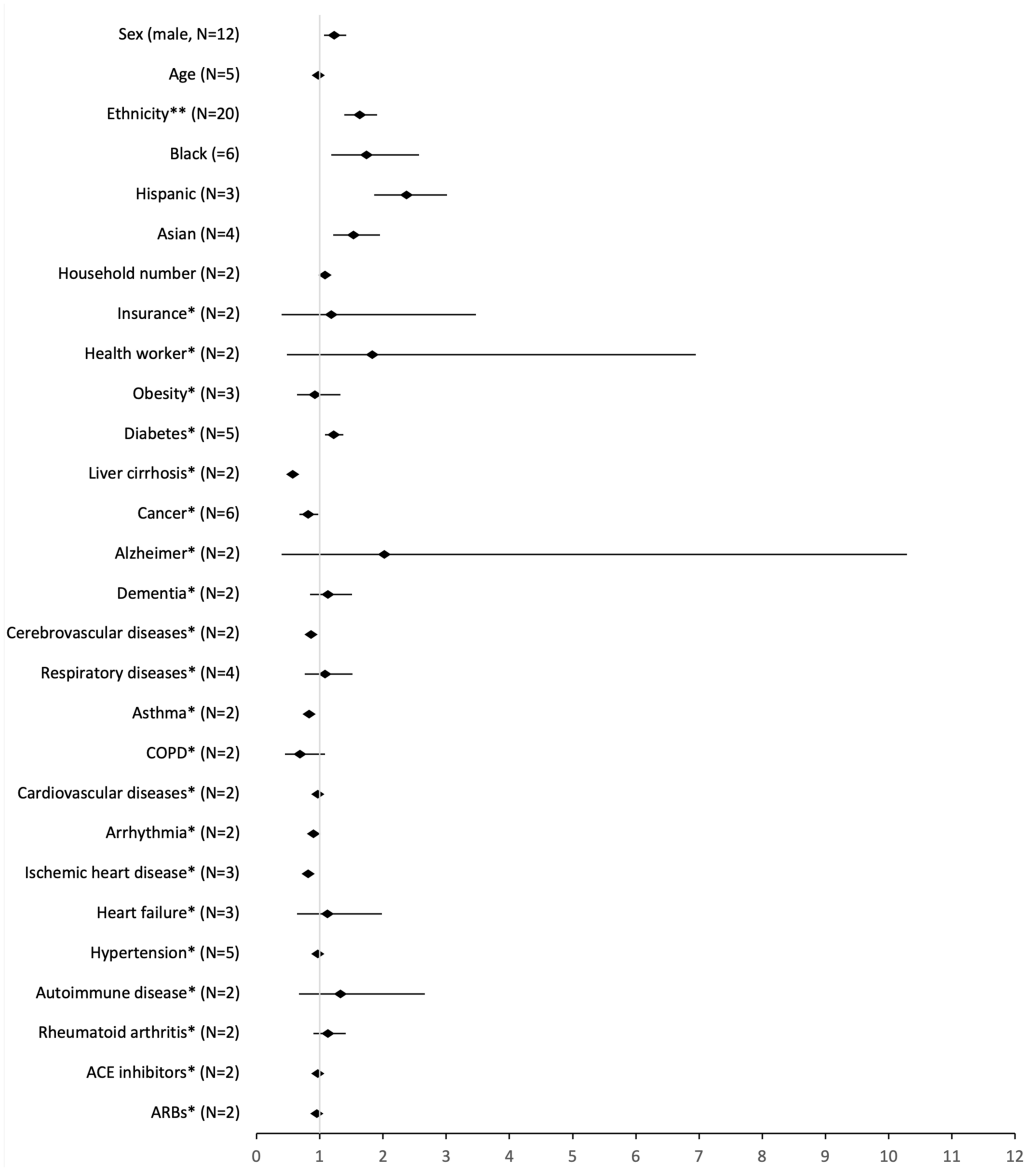
Pooled analysis of the risk factors for SARS-CoV-2 infection (high-income countries, 2020–2021). *reference: no; **reference: white.

We conducted a meta-analysis for 21 risk factors (Figure 2, detailed results in Supplementary File 5). Two of the factors were continuous variables: age and household number. Most were categorical variables. For sex and ethnicity, the reference was female and white, respectively. The remaining variables were classified as yes or no, presence or absence. In these cases, the reference was no/absence. The variables in this situation were: health worker, insurance, asthma, obesity, diabetes, hypertension, angiotensin-converting enzyme (ACE) inhibitors, angiotensin receptor blockers (ARBs), Alzheimer, dementia, chronic obstructive pulmonary disease (COPD), arrhythmia, ischemic heart disease, heart failure, liver cirrhosis, rheumatoid arthritis, cancer, cerebrovascular, respiratory and cardiovascular diseases. Variable cancer includes any diagnosis of cancer, and variable cerebrovascular diseases include stroke, transient cerebral ischemia and cerebral hemorrhages. The diseases included in each group are in Supplementary File 6.

Being a man (OR: 1.23, 95% CI: 1.97–1.42), of non-white ethnicity (OR: 1.63, 95% CI: 1.39–1.91), increasing household number (OR: 1.08, 95% CI: 1.06–1.10), or having diabetes (OR: 1.22, 95% CI: 1.08–1.37), were associated with an increased odds of getting SARS-CoV-2 infection. In contrast, having asthma (OR: 0.83, 95% CI: 0.75–0.92), ischemic heart disease (OR: 0.82, 95% CI: 0.74–0.91), cancer (OR: 0.82, 95% CI: 0.68–0.98), or cardiovascular diseases (OR: 0.92, 95% CI: 0.84–1.00) were associated with a decreased odds for the infection.

The sensitivity analysis for ethnicity without the category “Other” yielded results almost identical to the main analysis (OR: 1.70, 95% CI: 1.40–2.07). Comparing different ethnicities, Hispanics had a higher OR for SARS-CoV-2 infection (OR: 2.37, 95% CI: 1.86–3.01), followed by Black people (OR: 1.74, 95% CI: 1.18–2.57) and Asians (OR: 1.53, 95% CI: 1.21–1.95). The sensitivity analysis for type II diabetes yielded different results (OR: 1.08, 95% CI: 0.77–1.50) from the main analysis, not being a significant factor for the risk of infection. None of the sensitivity analyses for comorbidities were statistically significant.

All funnel plots suggested eventual publication bias, which is expected in a meta-analysis with observational studies (59).

## Discussion

This review aimed to synthesize the available evidence on risk factors associated with SARS-CoV-2 infection in high-income countries and quantify them. The high infection rate in high-income countries and the contradictory evidence found for some underlying diseases motivated this SR. Sex, ethnicity, household number and diabetes were associated with an increased risk of SARS-CoV-2 infection and asthma, ischemic heart disease, cancer and cardiovascular disease were associated with a decreased odds of infection.

Men showed a higher likelihood of becoming infected with SARS-CoV-2. This is consistent with records from other countries and meta-analyses related to SARS-CoV-2 infection (60, 61) and susceptibility trends for other respiratory viruses (62). Other studies suggest that this difference might be caused by biological and immune differences between females and males (63–65), namely neurological manifestations (66). Although there are several explanations for sex variations, the most common reason seems to be gender roles (60). A SR that specifically analyzed sex differences in COVID-19 (pooled prevalence men: 55.0, 51.4–56.6, *I*^2^ = 99.5%) states the differences found were due to the role and behaviors of men and women in the society (60). Detailing, more men are working in essential sectors and occupations that require them to continue being active, to work outside their homes and interact with other people even during lockdowns (e.g., manufacturing and sales, agriculture, food production and distribution, transportation, and security) (60). An independent initiative, to promote gender equality in health, stated that the number of cases between men and women vary with age and stay apparently balanced (67). This can support the behavior’s theory, as occupational issues also become less significant as people age. It is important to note that this report includes worldwide data, and the number of cases depends directly on the availability of tests. Data on testing disaggregated by sex is only available from a small number of countries, which makes it difficult to know if the case numbers suffer from ascertainment bias (67). This is also true for other respiratory viruses, like influenza, where it is difficult to ascertain the precise number of cases worldwide, for which a laboratory test is also necessary to confirm the disease, and because there is also insufficient data from less developed countries (62). Further studies are required to address underlying factors explaining such differences.

Non-white individuals also showed higher odds of getting infected, particularly Hispanic. Health determinants could explain this result, as ethnic minorities are commonly at the lower socioeconomic levels. Consequently, these populations tend to be over-represented in essential jobs with more contact with the public, living in worse neighborhoods or overcrowded houses, increasing the infection risk (68). This trend was consistent with literature from other studies, mainly from the United States of America and the United Kingdom (69). However, these results should be interpreted with caution because they are mainly from only two countries whose results might be specific to its social context (70). It is worth mentioning that studies often include a category “other,” not described in detail, which makes interpreting the results challenging since we cannot know which ethnicities are included (37, 39, 50, 53, 56). Nevertheless, our results add to the body of literature in this area.

We have identified several studies assessing pre-existing conditions with distinct results in our meta-analyses (36, 42, 44, 45, 47, 49–52, 56). From all the comorbidities analyzed, having a diabetes diagnosis was the only one with a higher chance of SARS-CoV-2 infection. A meta-analysis found that diabetes was the second most prevalent comorbidity in SARS-CoV-2 infected patients (9.7, 95% CI: 7.2–12.2%) (71). This disease could affect the immune system and weaken the immune response against SARS-CoV-2 infection (72) which is also affected by the nutritional uptake that is influenced by diabetes (73). Having asthma was identified as having lower odds of SARS-CoV-2 infection. An earlier literature review focused on the influence of this comorbidity in SARS-CoV-2 infection found no association between asthma and SARS-CoV-2 infection (74). This finding could suggest corticosteroids and bronchodilators, treatments for respiratory diseases, may reduce SARS-CoV-2 infection risk or reduce symptoms development leading to diagnosis (75). However, this has contrasting evidence (76, 77), being at the moment unclear the benefits and harms of respiratory disease treatments to the risk of COVID-19 infection.

Having a cancer diagnosis or a cardiovascular disease also showed lower odds for SARS-CoV-2 infection. However, there is evidence that cardiovascular diseases are important risk factors for respiratory viruses (78). Specifically related to cancer, there is evidence mentioning the weakened immune system of these patients (13, 79), and that regular visits to healthcare facilities for therapy may expose them to the virus (80). The high heterogeneity found between studies could be a reason for the apparent contradictory effect related to comorbidities.

Of the five comorbidities that showed significative ORs, four had protective results. To the best of our knowledge there is no underlying biological mechanism that explains this. Thus, similar to other studies, we hypothesize that these findings might be related to the evidence that people with comorbidities are more cautious toward their health, being more likely to avoid social gatherings, wear masks in situations where distancing is not an option and adhere to lockdown measures, possibly because they perceive their risk of being infected with SARS-CoV-2 as higher (81). That is, individuals with underlying conditions are unlikely to be less prone to SARS-CoV-2 infection, but their risk can be lowered through protective behaviors. Additionally, for asthma, our meta-analysis was performed with only two studies, where asthma was present in 21% (52) and 4% (44) of SARS-CoV-2 cases. However, more evidence is needed to ascertain the effect of the aforementioned comorbidities on SARS-CoV-2 infection risk.

Antihypertensors, namely ACE and ARB, were not significantly associated with SARS-CoV-2 infection, as other meta-analyses also indicate (82, 83). One of these studies analyzed the combined effect of these two medications (OR: 0.95, 95% CI: 0.89–1.02), showing that there is no evidence that this medication significantly increases the risk of infection (83). These results might be associated with SARS-CoV-2 transmission dynamics, which is mainly transmitted through the respiratory tract ACE2 receptors. There is no evidence to date reporting the expression of ACE2 receptors in lung tissue after ACEI/ARB treatment (83). This suggested reduction could not be confirmed in our analysis, which only included two articles for this variable.

Other risk factors, such as income, education, smoking status and drinking status, were reported in the included studies, but meta-analyses could not be performed due to heterogeneity of classification and analysis. These challenges understanding who has a higher risk of getting infected and what behaviors contribute to a higher risk of infection. Furthermore, the aforementioned factors had contradictory results in the individual studies that reported them, confirming the heterogeneity that could result in confusing guidelines to control the spreading and infection rate. In future studies on individual risk factors for SARS-CoV-2 infection, authors should analyze the variables more consistently, considering the published literature on the subject. For example, in one study, education was analyzed according to specific levels from the UK education system (42), challenging comparison with other international results. Additionally, it would be important for authors to provide more detailed information, as previously pointed out, in improving reporting initiatives (84).

Contextual factors could also have an important association with SARS-CoV-2 risk. However, since we excluded ecological studies, we only analyzed sociodemographic and behavioral factors. Although individual factors are important, the effect of contextual factors should also be assessed, i.e., where individuals live and/or work, type of transportation they use, and their access to health services. We urge authors to consider both factors in future analyses since analyzing them jointly strengthens SARS-CoV-2 research, providing a more comprehensive understanding of this disease (85). Although this systematic review is focused on the Alpha variant, new variants were emerging with an increasingly transmissibility and different patterns each time (86). Thus, would be important to replicate this type of studies for new variants to enhance SARS-CoV-2 epidemiologic surveillance.

There are several limitations to this study. First, we included studies whose outcome was only laboratory-confirmed SARS-CoV-2 infection through RT-PCR tests. This can potentially exclude a considerable volume of studies detected by another method. Thus, underestimating our results since severe cases, often associated with more risk factors, were not always laboratory confirmed. Additionally, in the early stages of the pandemic, RT-PCR tests mainly supported diagnosis. Thus, their scarcity meant that their use was directed toward healthcare professionals, residents and professionals in residential homes and symptomatic people. This is reported in some of the articles included, which may skew the results toward populations with higher risk. Although rapid antigen SARS-CoV-2 tests could reach more people, they were only available in late 2020 (87) or early 2021 (88), influencing the case definition between countries. RT-PCR tests remained the gold standard method of diagnosis during the study period, providing more consistent and reliable results. Additionally, restricting the systematic review only to high-income countries could also left out countries with high incidence and potentially with important information to the study of this disease. Another limitation is the rapid evolution of the SARS-CoV-2 virus, which results in the appearance of variants that differ in transmissibility, meaning that as new variants appear, risk factors may also change. Comorbidities can also pose another limitation because, in some studies, they were self-reported and it was unclear whether it was an acute or chronic illness. For this meta-analysis, we extracted the ORs of the most complete analyses, whose variables adjusted had some variation between studies. Values adjusted for the higher number of confounders tend to be closer to the real effect. Still, they can also increase heterogeneity between studies, which results should be interpreted with caution. The contradictory evidence found, namely for cancer and cardiovascular diseases, possibly due to inconsistent terminology describing the diseases and methodology used to extract data, highlights the challenging task of comprehending the true effect of the underlying risk factors for SARS-CoV-2 infection. Results for diabetes should be interpreted cautiously since articles for meta-analysis were included regarding the type of diabetes (I and II). We performed a sensitivity analysis with the articles analyzing the same type of diabetes (type II), which were only two articles. Another limitation could be related to vaccination against COVID-19 since vaccines started to be administrated in late 2020, during our study period. However, vaccine effectiveness against SARS-CoV-2 infection is lower than against severe COVID-19 (89). One study found that vaccine effectiveness against SARS-CoV-2 infection was lower among individuals with comorbidities than individuals without. Thus, it remains crucial to understand who is at higher risk for infection (90).

This review has several strengths since it is, to our knowledge, the first SR and comprehensive meta-analysis of risk factors for SARS-CoV-2 infection in high-income countries, thus adding important knowledge to the SARS-CoV-2 infection. The meta-analyses were conducted using methods that were most suited for the data extracted, considering the heterogeneity of the studies included. Choosing only one measure of effect for the meta-analysis ensured the homogeneity between studies and thus yielded more robust results. The comprehensive search strategy and the databases included, returning a high number of studies, also strengthens this study. The reliability of the study selection criteria was confirmed by double screening of included articles and by testing a random sample (10%) of the extraction form. The study quality was also verified with quality assessment tools there are robust and widely used in literature.

In conclusion, our study demonstrated that men, people of black ethnicity, increased household number, and having diabetes diagnosis were associated with an increased risk for SARS-CoV-2 infection. However, cardiovascular diseases, asthma and ischemic heart disease were shown to be protective factors for this disease. One of the limitations of this meta-analysis relates to the heterogeneity between studies. Thus, future studies should consider how variables are measured to improve comparison between studies and enable a more robust gathering of information from academics.

## Data availability statement

The original contributions presented in the study are included in the article/Supplementary material, further inquiries can be directed to the corresponding author.

## Author contributions

MM: Conceptualization, Formal analysis, Investigation, Methodology, Software, Writing – original draft, Writing – review & editing. SP: Data curation, Investigation, Validation, Writing – review & editing. PS: Conceptualization, Formal analysis, Investigation, Methodology, Supervision, Validation, Writing – review & editing. PA: Formal analysis, Methodology, Validation, Writing – review & editing. HD: Data curation, Software, Writing – review & editing. AL: Conceptualization, Formal analysis, Investigation, Methodology, Software, Supervision, Validation, Writing – review & editing.
