# Supplementary material for: Individual risk factors associated with SARS-CoV-2 infection during Alpha variant in high-income countries: a systematic review and meta-analysis
Source: Front Public Health. 2024 Jul 30;12:1367480. doi: 10.3389/fpubh.2024.1367480 (PMC11319152; doi:10.3389/fpubh.2024.1367480)
Supplement: Supplementary file 1 [file Data_Sheet_1.ZIP › SF2_Search Strategy COVID-19.docx]

| **Search strategy** |
| --- |
| 1. ‘COVID-19’ OR ‘sars-cov-2’ OR ‘Severe Acute Respiratory Syndrome Coronavirus 2’ OR ‘NCOV’ OR ‘2019 NCOV’ OR ‘new coronavirus’ OR ‘novel coronavirus disease’ [Title/Abstract] |
| 2. ‘COVID-19’ OR ‘sars-cov-2’ [MeSH Terms] |
| 3. 1 OR 2 |
| 4. ‘risk factors’ OR ‘determinants’ OR ‘characteristics associated’ OR ‘inequalities’ OR ‘factors associated’ [Title/Abstract] |
| 5. ‘risk factors’ OR ‘Social determinants of health’ OR ‘epidemiologic factor’ OR ‘Sociological Factors’ [MeSH Terms]) |
| 6. 4 OR 5 |
| 7. ‘Andorra’ OR ‘Antigua And Barbuda’ OR ‘Aruba’ OR ‘Australia’ OR ‘Austria’ OR ‘Bahamas’ OR ‘Bahrain’ OR ‘Barbados’ OR ‘Belgium’ OR ‘Bermuda’ OR ‘British Virgin Islands’ OR ‘Brunei’ OR ‘Canada’ OR ‘Cayman Islands’ OR ‘Channel Islands’ OR ‘Chile’ OR ‘Croatia’ OR ‘Curacao’ OR ‘Cyprus’ OR ‘Czech Republic’ OR ‘Denmark’ OR ‘Estonia’ OR ‘Faroe Islands’ OR ‘Finland’ OR ‘France’ OR ‘French Polynesia’ OR ‘Germany’ OR ‘Gibraltar’ OR ‘Greece’ OR ‘Greenland’ OR ‘Guam’ OR ‘Hong Kong’ OR ‘Hungary’ OR ‘Iceland’ OR ‘Ireland’ OR ‘Isle Of Man’ OR ‘Israel’ ‘Italy’ OR ‘Japan’ OR ‘Korea’ OR ‘Republic of Kuwait’ OR ‘Latvia’ OR ‘Liechtenstein’ OR ‘Lithuania’ OR ‘Luxembourg’ OR ‘Macau’ OR ‘Malta’ OR ‘Mauritius’ OR ‘Monaco’ OR ‘Nauru’ OR ‘Netherlands’ OR ‘New Caledonia’ OR ‘New Zealand’ OR ‘Northern Mariana Islands’ OR ‘Norway’ ‘Oman’ OR ‘Palau’ OR ‘Panama’ OR ‘Poland’ OR ‘Portugal’ OR ‘Puerto Rico’ OR ‘Qatar’ OR ‘Romania’ OR ‘San Marino’ OR ‘Saudi Arabia’ OR ‘Seychelles’ OR ‘Singapore’ OR ‘Sint Maarten’ OR ‘Slovak Republic’ OR ‘Slovakia’ OR ‘Slovenia’ OR ‘Spain’ OR ‘Saint Kitts and Nevis’ OR ‘Saint Martin’ OR ‘Sweden’ OR ‘Switzerland’ OR ‘Trinidad And Tobago’ OR ‘Turks And Caicos Islands’ OR ‘United Arab Emirates’ OR ‘United Kingdom’ OR ‘United States’ OR ‘Uruguay’ OR ‘Virgin Islands’ OR ‘Europe’ [Title/Abstract] |
| 8. ‘Andorra’ OR ‘Antigua and Barbuda’ OR ‘Aruba’ OR ‘Australia’ OR ‘Austria’ OR ‘Bahamas’ OR ‘Bahrain’ OR ‘Barbados’ OR ‘Belgium’ OR ‘Bermuda’ OR ‘British Virgin Islands’ OR ‘Brunei’ OR ‘Canada’ OR ‘West Indies’ OR ‘Channel Islands’ OR ‘Chile’ OR ‘Croatia’ OR ‘Curacao’ OR ‘Cyprus’ OR ‘Czech Republic’ OR ‘Denmark’ OR ‘Estonia’ OR ‘Finland’ OR ‘France’ OR ‘Polynesia’ OR ‘Germany’ OR ‘Gibraltar’ OR ‘Greece’ OR ‘Greenland’ OR ‘Guam’ OR ‘Hong Kong’ OR ‘Hungary’ OR ‘Iceland’ OR ‘Ireland’ OR ‘Israel’ ‘Italy’ OR ‘Japan’ OR ‘Republic of Korea’ OR ‘Kuwait’ OR ‘Latvia’ OR ‘Liechtenstein’ OR ‘Lithuania’ OR ‘Luxembourg’ OR ‘Macau’ OR ‘Malta’ OR ‘Mauritius’ OR ‘Monaco’ OR ‘Micronesia’ OR ‘Netherlands’ OR ‘New Caledonia’ OR ‘New Zealand’ OR ‘Norway’ OR ‘Oman’ OR ‘Palau’ OR ‘Panama’ OR ‘Poland’ OR ‘Portugal’ OR ‘Puerto Rico’ OR ‘Qatar’ OR ‘Romania’ OR ‘San Marino’ OR ‘Saudi Arabia’ OR ‘Seychelles’ OR ‘Singapore’ OR ‘Sint Maarten’ OR ‘Slovakia’ OR ‘Slovenia’ OR ‘Spain’ OR ‘Saint Kitts and Nevis’ OR ‘Sweden’ OR ‘Switzerland’ OR ‘Trinidad And Tobago’ OR ‘United Arab Emirates’ OR ‘United Kingdom’ OR ‘United States’ OR ‘Uruguay’ OR ‘United States Virgin Islands’ OR ‘Europe’ [MeSH Terms] |
| 9. 7 OR 8 |
| 10. 3 AND 6 AND 9 |
| **PubMed** (2020/1/1:2021/4/22) |
| (‘COVID-19’ [MeSH Terms] OR ‘COVID-19’ [Title/Abstract] OR ‘sars-cov-2’ [MeSH Terms] OR ‘sars-cov-2’ [Title/Abstract] OR ‘coronavirus’ [MeSH Terms] OR "Severe Acute Respiratory Syndrome Coronavirus 2"[ Title/Abstract] OR ‘NCOV’ [Title/Abstract] OR ‘2019 NCOV’ [Title/Abstract] OR ‘new coronavirus [Title/Abstract] OR ‘novel coronavirus disease’ [Title/Abstract]) AND (‘risk factors’ [MeSH Terms] OR ‘risk factors’ [Title/Abstract] OR ‘determinants’ [Title/Abstract] OR ‘Social determinants of health’ [MeSH Terms] OR ‘characteristics associated’ [Title/Abstract] OR ‘epidemiologic factor’ [MeSH Terms] OR ‘inequalities’ [Title/Abstract] OR ‘Sociological Factors’ [MeSH Terms] OR ‘factors associated’ [Title/Abstract]) AND (‘Andorra’[Title/Abstract] OR ‘Antigua And Barbuda’ [Title/Abstract] OR ‘Aruba’ [Title/Abstract] OR ‘Australia’ [Title/Abstract] OR ‘Austria’ [Title/Abstract] OR ‘Bahamas’ [Title/Abstract] OR ‘Bahrain’ [Title/Abstract] OR ‘Barbados’ [Title/Abstract] OR ‘Belgium’ [Title/Abstract] OR ‘Bermuda’ [Title/Abstract] OR ‘British Virgin Islands’ [Title/Abstract] OR ‘Brunei’ [Title/Abstract] OR ‘Canada’ [Title/Abstract] OR ‘Cayman Islands’ [Title/Abstract] OR ‘Channel Islands’ [Title/Abstract] OR ‘Chile’ [Title/Abstract] OR ‘Croatia’ [Title/Abstract] OR ‘Curacao’ [Title/Abstract] OR ‘Cyprus’ [Title/Abstract] OR ‘Czech Republic’ [Title/Abstract] OR ‘Denmark’ [Title/Abstract] OR ‘Estonia’ [Title/Abstract] OR ‘Faroe Islands’ [Title/Abstract] OR ‘Finland’ [Title/Abstract] OR ‘France’ [Title/Abstract] OR ‘French Polynesia’ [Title/Abstract] OR ‘Germany’ [Title/Abstract] OR ‘Gibraltar’ [Title/Abstract] OR ‘Greece’ [Title/Abstract] OR ‘Greenland’ [Title/Abstract] OR ‘Guam’ [Title/Abstract] OR ‘Hong Kong’ [Title/Abstract] OR ‘Hungary’ [Title/Abstract] OR ‘Iceland’ [Title/Abstract] OR ‘Ireland’ [Title/Abstract] OR ‘Isle Of Man’ [Title/Abstract] OR ‘Israel’ ‘Italy’ [Title/Abstract] OR ‘Japan’ [Title/Abstract] OR ‘Korea’ [Title/Abstract] OR ‘Republic of Kuwait’ [Title/Abstract] OR ‘Latvia’ [Title/Abstract] OR ‘Liechtenstein’ [Title/Abstract] OR ‘Lithuania’ [Title/Abstract] OR ‘Luxembourg’ [Title/Abstract] OR ‘Macau’ [Title/Abstract] OR ‘Malta’ [Title/Abstract] OR ‘Mauritius’ [Title/Abstract] OR ‘Monaco’ [Title/Abstract] OR ‘Nauru’ [Title/Abstract] OR ‘Netherlands’ [Title/Abstract] OR ‘New Caledonia’ [Title/Abstract] OR ‘New Zealand’ [Title/Abstract] OR ‘Northern Mariana Islands’ [Title/Abstract] OR ‘Norway’ [Title/Abstract] ‘Oman’ [Title/Abstract] OR ‘Palau’ [Title/Abstract] OR ‘Panama’ [Title/Abstract] OR ‘Poland’ [Title/Abstract] OR ‘Portugal’ [Title/Abstract] OR ‘Puerto Rico’ [Title/Abstract] OR ‘Qatar’ [Title/Abstract] OR ‘Romania’ [Title/Abstract] OR ‘San Marino’ [Title/Abstract] OR ‘Saudi Arabia’ [Title/Abstract] OR ‘Seychelles’ [Title/Abstract] OR ‘Singapore’ [Title/Abstract] OR ‘Sint Maarten’ [Title/Abstract] OR ‘Slovak Republic’ [Title/Abstract] OR ‘Slovakia’ [Title/Abstract] OR ‘Slovenia’ [Title/Abstract] OR ‘Spain’ [Title/Abstract] OR ‘Saint Kitts and Nevis’ [Title/Abstract] OR ‘Saint Martin’ [Title/Abstract] OR ‘Sweden’ [Title/Abstract] OR ‘Switzerland’ [Title/Abstract] OR ‘Trinidad And Tobago’ [Title/Abstract] OR ‘Turks And Caicos Islands’ [Title/Abstract] OR ‘United Arab Emirates’ [Title/Abstract] OR ‘United Kingdom’ [Title/Abstract] OR ‘United States’ [Title/Abstract] OR ‘Uruguay’ [Title/Abstract] OR ‘Virgin Islands’ [Title/Abstract] OR ‘Andorra’ [MeSH Terms] OR ‘Antigua and Barbuda’ [MeSH Terms] OR ‘Aruba’ [MeSH Terms] OR ‘Australia’ [MeSH Terms] OR ‘Austria’ [MeSH Terms] OR ‘Bahamas’ [MeSH Terms] OR ‘Bahrain’ [MeSH Terms] OR ‘Barbados’ [MeSH Terms] OR ‘Belgium’ [MeSH Terms] OR ‘Bermuda’ [MeSH Terms] OR ‘British Virgin Islands’ [MeSH Terms] OR ‘Brunei’ [MeSH Terms] OR ‘Canada’ [MeSH Terms] OR ‘West Indies’ [MeSH Terms] OR ‘Channel Islands’ [MeSH Terms] OR ‘Chile’ [MeSH Terms] OR ‘Croatia’ [MeSH Terms] OR ‘Curacao’ [MeSH Terms] OR ‘Cyprus’ [MeSH Terms] OR ‘Czech Republic’ [MeSH Terms] OR ‘Denmark’ [MeSH Terms] OR ‘Estonia’ [MeSH Terms] OR ‘Finland’ [MeSH Terms] OR ‘France’ [MeSH Terms] OR ‘Polynesia’ [MeSH Terms] OR ‘Germany’ [MeSH Terms] OR ‘Gibraltar’ [MeSH Terms] OR ‘Greece’ [MeSH Terms] OR ‘Greenland’ [MeSH Terms] OR ‘Guam’ [MeSH Terms] OR ‘Hong Kong’ [MeSH Terms] OR ‘Hungary’ [MeSH Terms] OR ‘Iceland’ [MeSH Terms] OR ‘Ireland’ [MeSH Terms] OR ‘Israel’ ‘Italy’ [MeSH Terms] OR ‘Japan’ [MeSH Terms] OR ‘Republic of Korea’ [MeSH Terms] OR ‘Kuwait’ [MeSH Terms] OR ‘Latvia’ [MeSH Terms] OR ‘Liechtenstein’ [MeSH Terms] OR ‘Lithuania’ [MeSH Terms] OR ‘Luxembourg’ [MeSH Terms] OR ‘Macau’ [MeSH Terms] OR ‘Malta’ [MeSH Terms] OR ‘Mauritius’ [MeSH Terms] OR ‘Monaco’ [MeSH Terms] OR ‘Micronesia’ [MeSH Terms] OR ‘Netherlands’ [MeSH Terms] OR ‘New Caledonia’ [MeSH Terms] OR ‘New Zealand’ [MeSH Terms] OR ‘Norway’ [MeSH Terms] OR ‘Oman’ [MeSH Terms] OR ‘Palau’ [MeSH Terms] OR ‘Panama’ [MeSH Terms] OR ‘Poland’ [MeSH Terms] OR ‘Portugal’ [MeSH Terms] OR ‘Puerto Rico’ [MeSH Terms] OR ‘Qatar’ [MeSH Terms] OR ‘Romania’ [MeSH Terms] OR ‘San Marino’ [MeSH Terms] OR ‘Saudi Arabia’ [MeSH Terms] OR ‘Seychelles’ [MeSH Terms] OR ‘Singapore’ [MeSH Terms] OR ‘Sint Maarten’ [MeSH Terms] OR ‘Slovakia’ [MeSH Terms] OR ‘Slovenia’ [MeSH Terms] OR ‘Spain’ [MeSH Terms] OR ‘Saint Kitts and Nevis’ [MeSH Terms] OR ‘Sweden’ [MeSH Terms] OR ‘Switzerland’ [MeSH Terms] OR ‘Trinidad And Tobago’ [MeSH Terms] OR ‘United Arab Emirates’ [MeSH Terms] OR ‘United Kingdom’ [MeSH Terms] OR ‘United States’ [MeSH Terms] OR ‘Uruguay’ [MeSH Terms] OR ‘United States Virgin Islands’ [MeSH Terms] OR Europe [MeSH Terms] OR Europe [Title/Abstract]) |
| **Web of Science** |
| 1. (AB=('COVID-19' OR ‘sars-cov-2’ OR ‘Severe Acute Respiratory Syndrome Coronavirus 2’ OR ‘NCOV’ OR ‘2019 NCOV’ OR ‘new coronavirus’ OR ‘novel coronavirus disease’) |
| OR |
| TI=('COVID-19' OR ‘sars-cov-2’ OR ‘Severe Acute Respiratory Syndrome Coronavirus 2’ OR ‘NCOV’ OR ‘2019 NCOV’ OR ‘new coronavirus’ OR ‘novel coronavirus disease’)) |
| AND (AB=(‘risk factors’ OR ‘determinants’ OR ‘characteristics associated’ OR ‘inequalities’ OR ‘factors associated’ OR ‘Social determinants of health’ OR ‘epidemiologic factor’ OR ‘Sociological Factors’) |
| OR TI=(‘risk factors’ OR ‘determinants’ OR ‘characteristics associated’ OR ‘inequalities’ OR ‘factors associated’ OR ‘Social determinants of health’ OR ‘epidemiologic factor’ OR ‘Sociological Factors’)) |
| AND |
| (AB=(‘Andorra’ OR ‘Antigua And Barbuda’ OR ‘Aruba’ OR ‘Australia’ OR ‘Austria’ OR ‘Bahamas’ OR ‘Bahrain’ OR ‘Barbados’ OR ‘Belgium’ OR ‘Bermuda’ OR ‘British Virgin Islands’ OR ‘Brunei’ OR ‘Canada’ OR ‘Cayman Islands’ OR ‘Channel Islands’ OR ‘Chile’ OR ‘Croatia’ OR ‘Curacao’ OR ‘Cyprus’ OR ‘Czech Republic’ OR ‘Denmark’ OR ‘Estonia’ OR ‘Faroe Islands’ OR ‘Finland’ OR ‘France’ OR ‘French Polynesia’ OR ‘Germany’ OR ‘Gibraltar’ OR ‘Greece’ OR ‘Greenland’ OR ‘Guam’ OR ‘Hong Kong’ OR ‘Hungary’ OR ‘Iceland’ OR ‘Ireland’ OR ‘Isle Of Man’ OR ‘Israel’ ‘Italy’ OR ‘Japan’ OR ‘Korea’ OR ‘Republic of Kuwait’ OR ‘Latvia’ OR ‘Liechtenstein’ OR ‘Lithuania’ OR ‘Luxembourg’ OR ‘Macau’ OR ‘Malta’ OR ‘Mauritius’ OR ‘Monaco’ OR ‘Nauru’ OR ‘Netherlands’ OR ‘New Caledonia’ OR ‘New Zealand’ OR ‘Northern Mariana Islands’ OR ‘Norway’ ‘Oman’ OR ‘Palau’ OR ‘Panama’ OR ‘Poland’ OR ‘Portugal’ OR ‘Puerto Rico’ OR ‘Qatar’ OR ‘Romania’ OR ‘San Marino’ OR ‘Saudi Arabia’ OR ‘Seychelles’ OR ‘Singapore’ OR ‘Sint Maarten’ OR ‘Slovak Republic’ OR ‘Slovakia’ OR ‘Slovenia’ OR ‘Spain’ OR ‘Saint Kitts and Nevis’ OR ‘Saint Martin’ OR ‘Sweden’ OR ‘Switzerland’ OR ‘Trinidad And Tobago’ OR ‘Turks And Caicos Islands’ OR ‘United Arab Emirates’ OR ‘United Kingdom’ OR ‘United States’ OR ‘Uruguay’ OR ‘Virgin Islands’ OR ‘Europe’) OR TI=(‘Andorra’ OR ‘Antigua And Barbuda’ OR ‘Aruba’ OR ‘Australia’ OR ‘Austria’ OR ‘Bahamas’ OR ‘Bahrain’ OR ‘Barbados’ OR ‘Belgium’ OR ‘Bermuda’ OR ‘British Virgin Islands’ OR ‘Brunei’ OR ‘Canada’ OR ‘Cayman Islands’ OR ‘Channel Islands’ OR ‘Chile’ OR ‘Croatia’ OR ‘Curacao’ OR ‘Cyprus’ OR ‘Czech Republic’ OR ‘Denmark’ OR ‘Estonia’ OR ‘Faroe Islands’ OR ‘Finland’ OR ‘France’ OR ‘French Polynesia’ OR ‘Germany’ OR ‘Gibraltar’ OR ‘Greece’ OR ‘Greenland’ OR ‘Guam’ OR ‘Hong Kong’ OR ‘Hungary’ OR ‘Iceland’ OR ‘Ireland’ OR ‘Isle Of Man’ OR ‘Israel’ ‘Italy’ OR ‘Japan’ OR ‘Korea’ OR ‘Republic of Kuwait’ OR ‘Latvia’ OR ‘Liechtenstein’ OR ‘Lithuania’ OR ‘Luxembourg’ OR ‘Macau’ OR ‘Malta’ OR ‘Mauritius’ OR ‘Monaco’ OR ‘Nauru’ OR ‘Netherlands’ OR ‘New Caledonia’ OR ‘New Zealand’ OR ‘Northern Mariana Islands’ OR ‘Norway’ ‘Oman’ OR ‘Palau’ OR ‘Panama’ OR ‘Poland’ OR ‘Portugal’ OR ‘Puerto Rico’ OR ‘Qatar’ OR ‘Romania’ OR ‘San Marino’ OR ‘Saudi Arabia’ OR ‘Seychelles’ OR ‘Singapore’ OR ‘Sint Maarten’ OR ‘Slovak Republic’ OR ‘Slovakia’ OR ‘Slovenia’ OR ‘Spain’ OR ‘Saint Kitts and Nevis’ OR ‘Saint Martin’ OR ‘Sweden’ OR ‘Switzerland’ OR ‘Trinidad And Tobago’ OR ‘Turks And Caicos Islands’ OR ‘United Arab Emirates’ OR ‘United Kingdom’ OR ‘United States’ OR ‘Uruguay’ OR ‘Virgin Islands’ OR ‘Europe’)) |
| 2. DOP=(2020-01-01/2021-05-31) |
| 3. #1 AND #2 |
| **MedrXiv** |
| (COVID-19 OR Sars-cov-2) AND (risk factors OR determinants OR characteristics associated OR factors associated)”, posted between "01 Jan, 2020 and 31 May, 2021" |

(AB=('COVID-19' OR ‘sars-cov-2’ OR ‘Severe Acute Respiratory Syndrome Coronavirus 2’ OR ‘NCOV’ OR ‘2019 NCOV’ OR ‘new coronavirus’ OR ‘novel coronavirus disease’)

OR

TI=('COVID-19' OR ‘sars-cov-2’ OR ‘Severe Acute Respiratory Syndrome Coronavirus 2’ OR ‘NCOV’ OR ‘2019 NCOV’ OR ‘new coronavirus’ OR ‘novel coronavirus disease’))

AND (AB=(‘risk factors’ OR ‘determinants’ OR ‘characteristics associated’ OR ‘inequalities’ OR ‘factors associated’ OR ‘Social determinants of health’ OR ‘epidemiologic factor’ OR ‘Sociological Factors’)

OR TI=(‘risk factors’ OR ‘determinants’ OR ‘characteristics associated’ OR ‘inequalities’ OR ‘factors associated’ OR ‘Social determinants of health’ OR ‘epidemiologic factor’ OR ‘Sociological Factors’))

AND

(AB=(‘Andorra’ OR ‘Antigua And Barbuda’ OR ‘Aruba’ OR ‘Australia’ OR ‘Austria’ OR ‘Bahamas’ OR ‘Bahrain’ OR ‘Barbados’ OR ‘Belgium’ OR ‘Bermuda’ OR ‘British Virgin Islands’ OR ‘Brunei’ OR ‘Canada’ OR ‘Cayman Islands’ OR ‘Channel Islands’ OR ‘Chile’ OR ‘Croatia’ OR ‘Curacao’ OR ‘Cyprus’ OR ‘Czech Republic’ OR ‘Denmark’ OR ‘Estonia’ OR ‘Faroe Islands’ OR ‘Finland’ OR ‘France’ OR ‘French Polynesia’ OR ‘Germany’ OR ‘Gibraltar’ OR ‘Greece’ OR ‘Greenland’ OR ‘Guam’ OR ‘Hong Kong’ OR ‘Hungary’ OR ‘Iceland’ OR ‘Ireland’ OR ‘Isle Of Man’ OR ‘Israel’ ‘Italy’ OR ‘Japan’ OR ‘Korea’ OR ‘Republic of Kuwait’ OR ‘Latvia’ OR ‘Liechtenstein’ OR ‘Lithuania’ OR ‘Luxembourg’ OR ‘Macau’ OR ‘Malta’ OR ‘Mauritius’ OR ‘Monaco’ OR ‘Nauru’ OR ‘Netherlands’ OR ‘New Caledonia’ OR ‘New Zealand’ OR ‘Northern Mariana Islands’ OR ‘Norway’ ‘Oman’ OR ‘Palau’ OR ‘Panama’ OR ‘Poland’ OR ‘Portugal’ OR ‘Puerto Rico’ OR ‘Qatar’ OR ‘Romania’ OR ‘San Marino’ OR ‘Saudi Arabia’ OR ‘Seychelles’ OR ‘Singapore’ OR ‘Sint Maarten’ OR ‘Slovak Republic’ OR ‘Slovakia’ OR ‘Slovenia’ OR ‘Spain’ OR ‘Saint Kitts and Nevis’ OR ‘Saint Martin’ OR ‘Sweden’ OR ‘Switzerland’ OR ‘Trinidad And Tobago’ OR ‘Turks And Caicos Islands’ OR ‘United Arab Emirates’ OR ‘United Kingdom’ OR ‘United States’ OR ‘Uruguay’ OR ‘Virgin Islands’ OR ‘Europe’) OR TI=(‘Andorra’ OR ‘Antigua And Barbuda’ OR ‘Aruba’ OR ‘Australia’ OR ‘Austria’ OR ‘Bahamas’ OR ‘Bahrain’ OR ‘Barbados’ OR ‘Belgium’ OR ‘Bermuda’ OR ‘British Virgin Islands’ OR ‘Brunei’ OR ‘Canada’ OR ‘Cayman Islands’ OR ‘Channel Islands’ OR ‘Chile’ OR ‘Croatia’ OR ‘Curacao’ OR ‘Cyprus’ OR ‘Czech Republic’ OR ‘Denmark’ OR ‘Estonia’ OR ‘Faroe Islands’ OR ‘Finland’ OR ‘France’ OR ‘French Polynesia’ OR ‘Germany’ OR ‘Gibraltar’ OR ‘Greece’ OR ‘Greenland’ OR ‘Guam’ OR ‘Hong Kong’ OR ‘Hungary’ OR ‘Iceland’ OR ‘Ireland’ OR ‘Isle Of Man’ OR ‘Israel’ ‘Italy’ OR ‘Japan’ OR ‘Korea’ OR ‘Republic of Kuwait’ OR ‘Latvia’ OR ‘Liechtenstein’ OR ‘Lithuania’ OR ‘Luxembourg’ OR ‘Macau’ OR ‘Malta’ OR ‘Mauritius’ OR ‘Monaco’ OR ‘Nauru’ OR ‘Netherlands’ OR ‘New Caledonia’ OR ‘New Zealand’ OR ‘Northern Mariana Islands’ OR ‘Norway’ ‘Oman’ OR ‘Palau’ OR ‘Panama’ OR ‘Poland’ OR ‘Portugal’ OR ‘Puerto Rico’ OR ‘Qatar’ OR ‘Romania’ OR ‘San Marino’ OR ‘Saudi Arabia’ OR ‘Seychelles’ OR ‘Singapore’ OR ‘Sint Maarten’ OR ‘Slovak Republic’ OR ‘Slovakia’ OR ‘Slovenia’ OR ‘Spain’ OR ‘Saint Kitts and Nevis’ OR ‘Saint Martin’ OR ‘Sweden’ OR ‘Switzerland’ OR ‘Trinidad And Tobago’ OR ‘Turks And Caicos Islands’ OR ‘United Arab Emirates’ OR ‘United Kingdom’ OR ‘United States’ OR ‘Uruguay’ OR ‘Virgin Islands’ OR ‘Europe’))
