# Supplementary material for: Individual risk factors associated with SARS-CoV-2 infection during Alpha variant in high-income countries: a systematic review and meta-analysis
Source: Front Public Health. 2024 Jul 30;12:1367480. doi: 10.3389/fpubh.2024.1367480 (PMC11319152; doi:10.3389/fpubh.2024.1367480)
Supplement: Supplementary file 1 [file Data_Sheet_1.ZIP › SF4_Table QA.docx]

Table S4. Quality assessment by study design.

| Cohort | | | | | | | | | | | | | | | | | | | | | | | |
| --- | --- | --- | --- | --- | --- | --- | --- | --- | --- | --- | --- | --- | --- | --- | --- | --- | --- | --- | --- | --- | --- | --- | --- |
| First author, year of publication | | | Representativeness of the exposed cohort | | | Selection of the non exposed cohort | | Ascertainment of exposure | | | Demonstration that outcome of interest was not present at start of study | | | | Comparability of cohorts on the basis of the design or analysis | | | Assessment of outcome | | Was follow-up long enough for outcomes to occur | | Total* | |
| **﻿Yizhou Yu ^et al^, 2021** | | |  | | |  | |  | | |  | | | |  | | |  | |  | | 7 | |
| **﻿Sachin J Shah ^et al^, 2020** | | |  | | |  | |  | | |  | | | |  | | |  | |  | | 5 | |
| **﻿Claire L. Niedzwiedz ^et al^, 2020** | | |  | | |  | |  | | |  | | | |  | | |  | |  | | 7 | |
| **﻿Xiude Fan ^et al^, 2020** | | |  | | |  | |  | | |  | | | |  | | |  | |  | | 7 | |
| **﻿﻿Sara J. Cromer ^et al^, 2020** | | |  | | |  | |  | | |  | | | |  | | |  | |  | | 8 | |
| **﻿﻿Jose L Pablos ^et al^, 2020** | | |  | | |  | |  | | |  | | | |  | | |  | |  | | 5 | |
| **﻿﻿Altug Didikoglu ^et al^, 2021** | | |  | | |  | |  | | |  | | | |  | | |  | |  | | 5 | |
| **Angel Vila- Córcoles ^et al^, 2020** | | |  | | |  | |  | | |  | | | |  | | |  | |  | | 8 | |
| **﻿Zahra Raisi‑Estabragh ^et al^, 2021** | | |  | | |  | |  | | |  | | | |  | | |  | |  | | 5 | |
| **﻿﻿Rohini Mathur ^et al^, 2021** | | |  | | |  | |  | | |  | | | |  | | |  | |  | | 7 | |
| **Frederick K Ho ^et al^, 2020** | | |  | | |  | |  | | |  | | | |  | | |  | |  | | 8 | |
| **Seung Won Lee ^et al^, 2020** | | |  | | |  | |  | | |  | | | |  | | |  | |  | | 8 | |
| **Harmony R. Reynolds ^et al^, 2020** | | |  | | |  | |  | | |  | | | |  | | |  | |  | | 7 | |
| Case-control | | | | | | | | | | | | | | | | | | | | | | | |
| First author, year of publication | Is the case definition adequate? | | | Representativeness of the cases | | | Selection of Controls | | | Definition of Controls | | Comparability of cases and controls on the basis of the design or analysis | | Ascertainment of exposure | | | Same method of ascertainment for cases and controls | | | | Non-Response rate | Total* | |
| **﻿Giuseppe Mancia ^et al^, 2020** |  | | |  | | |  | | |  | |  | |  | | |  | | | |  | 8 | |
| **﻿Ariel Israel ^et al^, 2020** |  | | |  | | |  | | |  | |  | |  | | |  | | | |  | 8 | |
| **﻿﻿Bing Zhang ^et al^, 2021** |  | | |  | | |  | | |  | |  | |  | | |  | | | |  | 7 | |
| **﻿Kuan-Han H. Wu ^et al^, 2021** |  | | |  | | |  | | |  | |  | |  | | |  | | | |  | 5 | |
| **﻿﻿Wonjun Ji ^et al^, 2020** |  | | |  | | |  | | |  | |  | |  | | |  | | | |  | 7 | |
| **﻿﻿Marc Chadeau-Hyam ^et al^, 2020** |  | | |  | | |  | | |  | |  | |  | | |  | | | |  | 6 | |
| **﻿﻿Eyrun F. Kjetland ^et al^, 2020** |  | | |  | | |  | | |  | |  | |  | | |  | | | |  | 5 | |
| **Jeongkuk Seo, 2020** |  | | |  | | |  | | |  | |  | |  | | |  | | | |  | 8 | |
| Analytical Cross Sectional | | | | | | | | | | | | | | | | | | | | | | | |
| First author, year of publication | | Were the criteria for inclusion in the sample clearly defined? | | | Were the study subjects and the setting described in detail? | Was the exposure measured in a valid and reliable way? | | | Were objective, standard criteria used for measurement of the condition? | | | | Were confounding factors identified? | | | Were strategies to deal with confounding factors stated? | Were the outcomes measured in a valid and reliable way? | | Was appropriate statistical analysis used? | | | | Total |
| **Farhaan S. Vahidy ^et al^, 2021** | |  | | |  |  | | |  | | | |  | | |  |  | |  | | | | 6 |
| **﻿Farhaan S Vahidy ^et al^, 2020** | |  | | |  |  | | |  | | | |  | | |  |  | |  | | | | 5 |
| **﻿L. Silvia Muñoz-Price ^et al^, 2020** | |  | | |  |  | | |  | | | |  | | |  |  | |  | | | | 8 |
| **﻿Ehab Hamed ^et al^, 2020** | |  | | |  |  | | |  | | | |  | | |  |  | |  | | | | 6 |
| **﻿Seon Cheol Park ^et al^, 2021** | |  | | |  |  | | |  | | | |  | | |  |  | |  | | | | 6 |
| **﻿Leonard E Egede ^et al^, 2020** | |  | | |  |  | | |  | | | |  | | |  |  | |  | | | | 8 |

Legend: green: information provided was complete or sufficient; red: no information; yellow: unclear information; grey: not applicable.
