# Supplementary material for: Individual risk factors associated with SARS-CoV-2 infection during Alpha variant in high-income countries: a systematic review and meta-analysis
Source: Front Public Health. 2024 Jul 30;12:1367480. doi: 10.3389/fpubh.2024.1367480 (PMC11319152; doi:10.3389/fpubh.2024.1367480)
Supplement: Supplementary file 1 [file Data_Sheet_1.ZIP › SF5_ Detailed meta-analysis and sensivity analysis.docx]

**Sex**


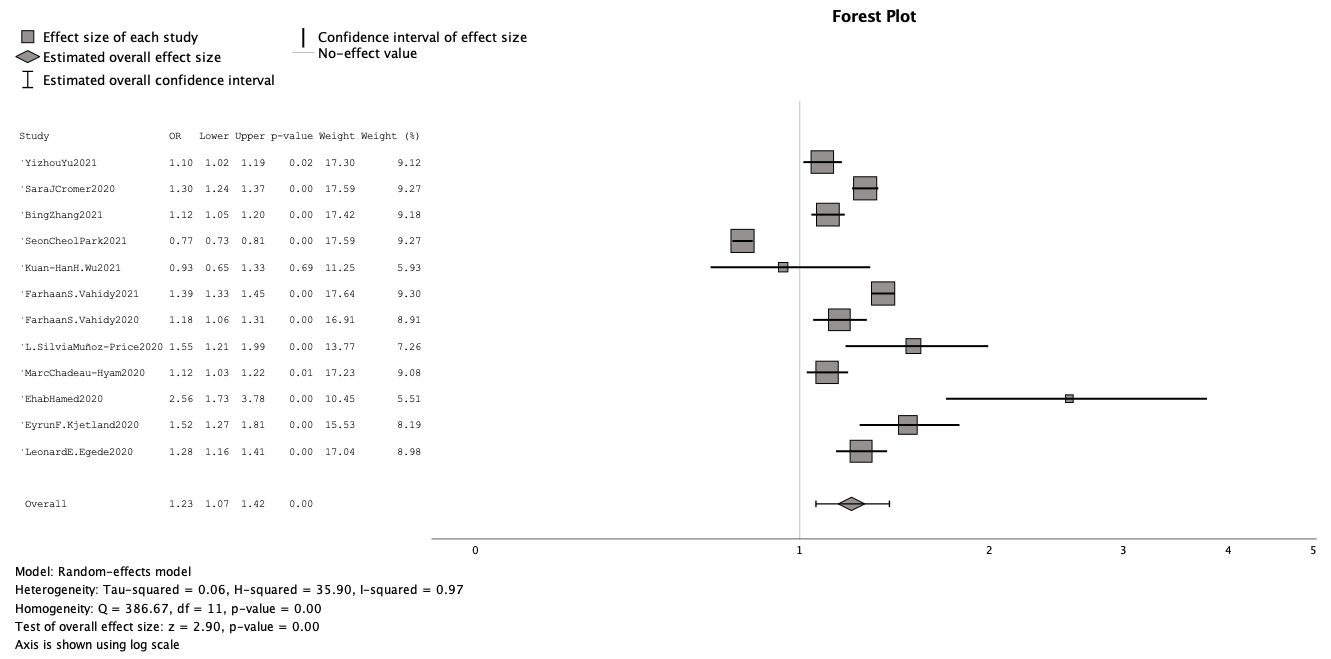


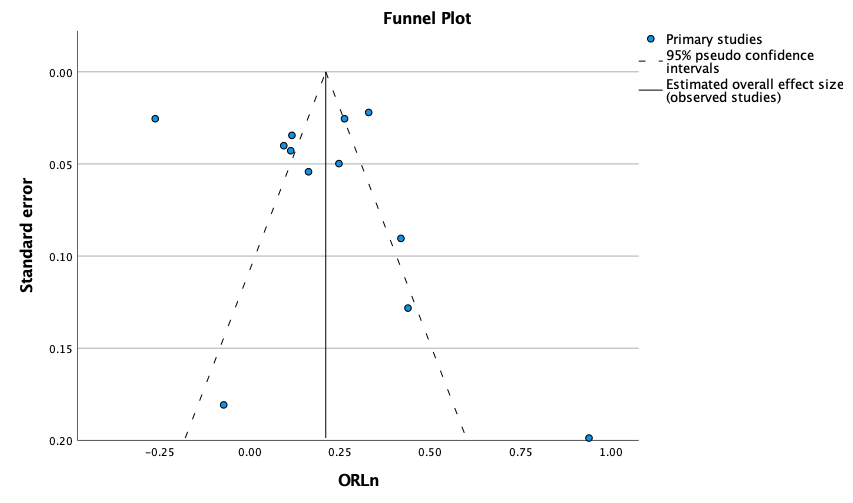


**Age**


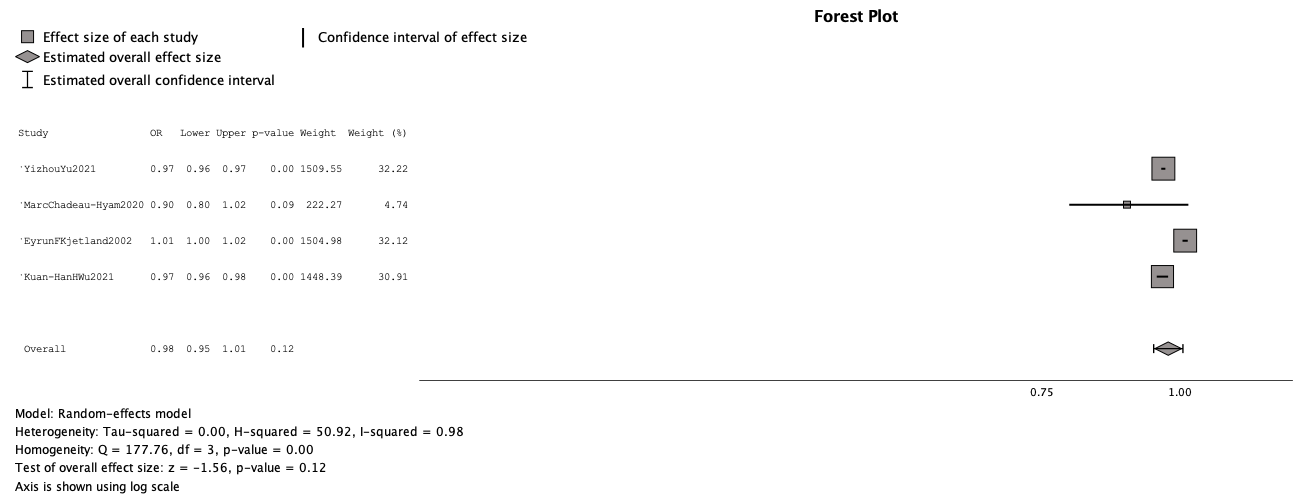


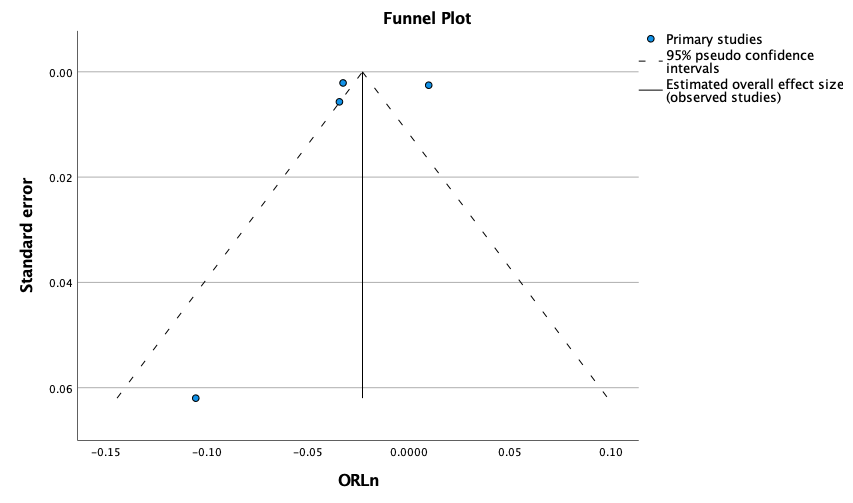


**Ethnicity**


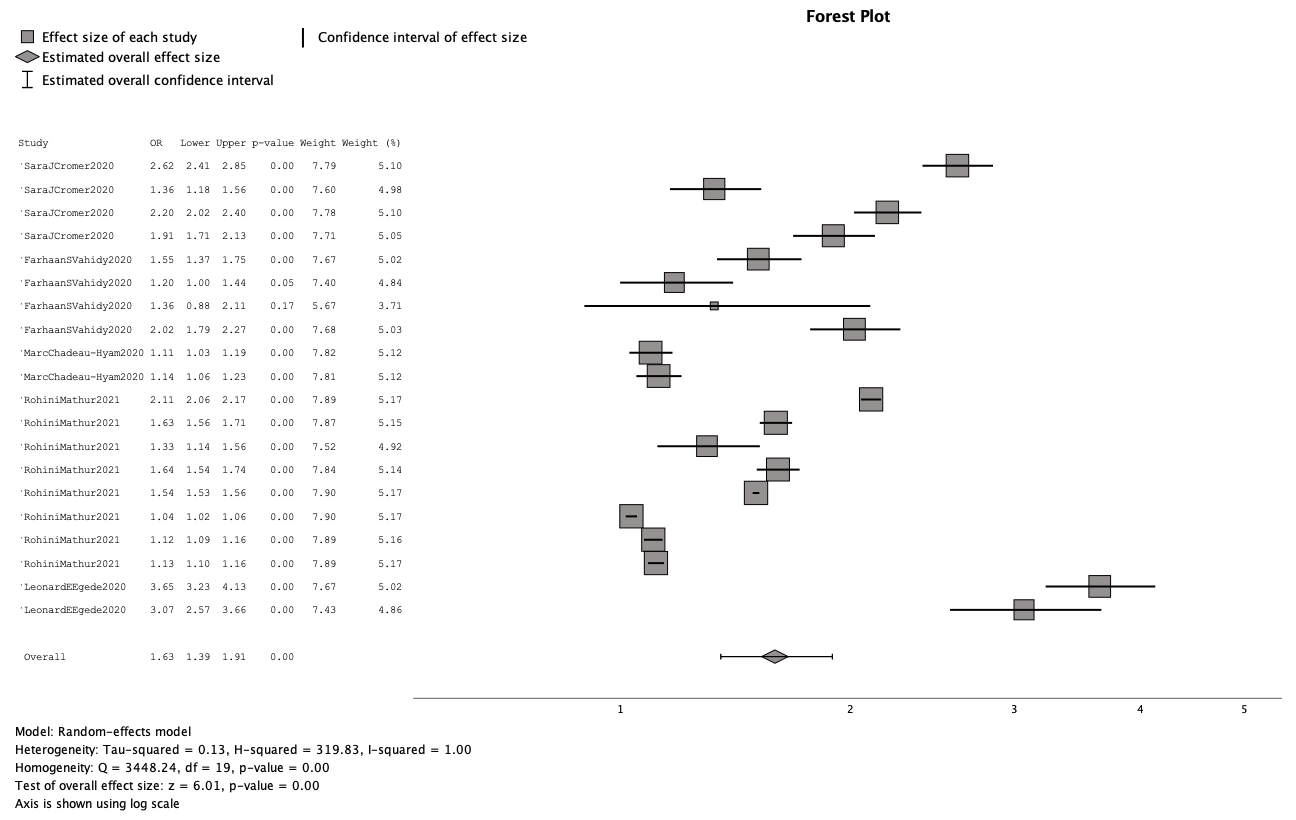


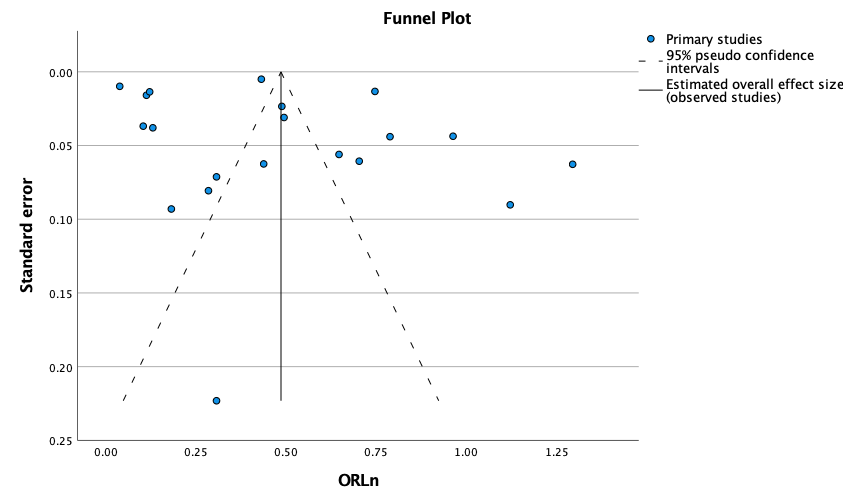


**Ethnicity** (without Rohiny Mathur 2021)


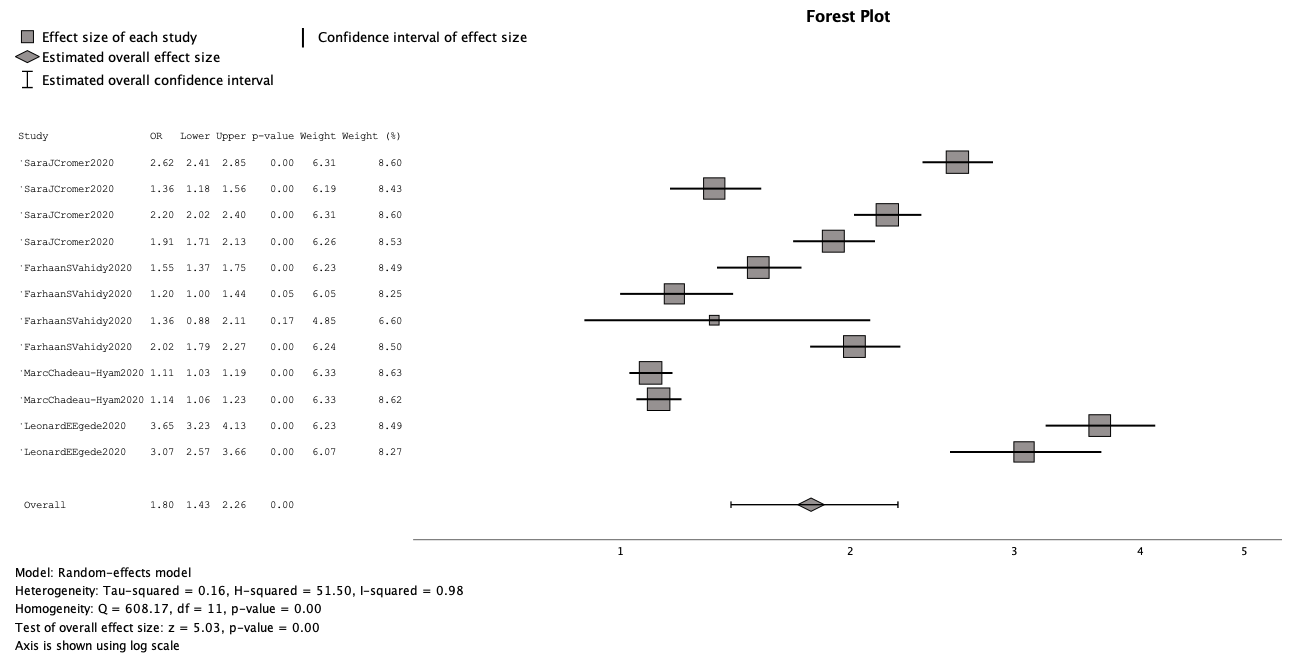


**
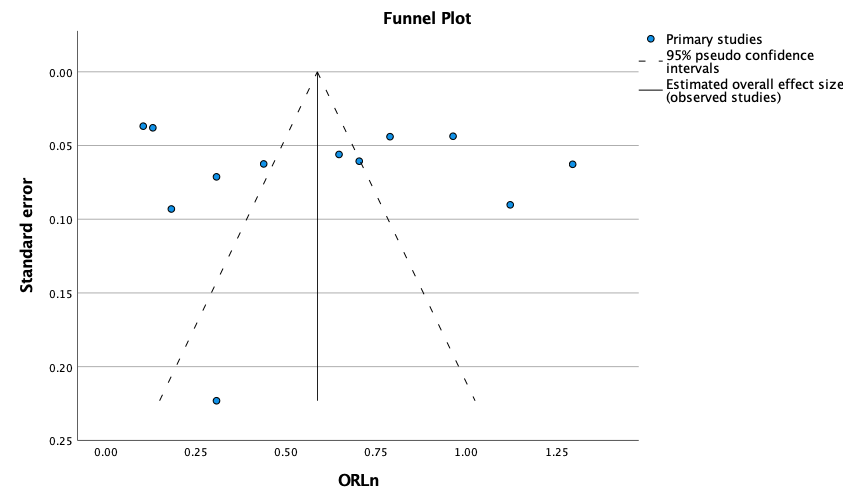
**

**Ethnicity** (without “Other”)


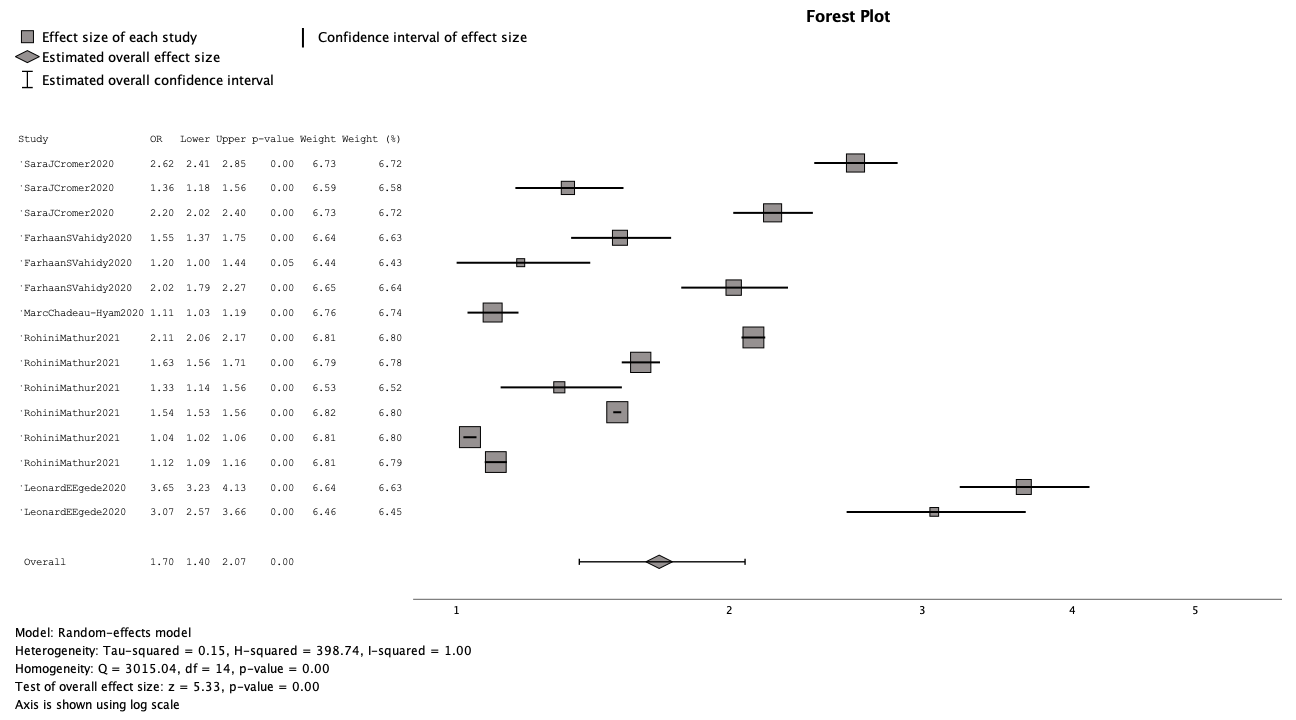


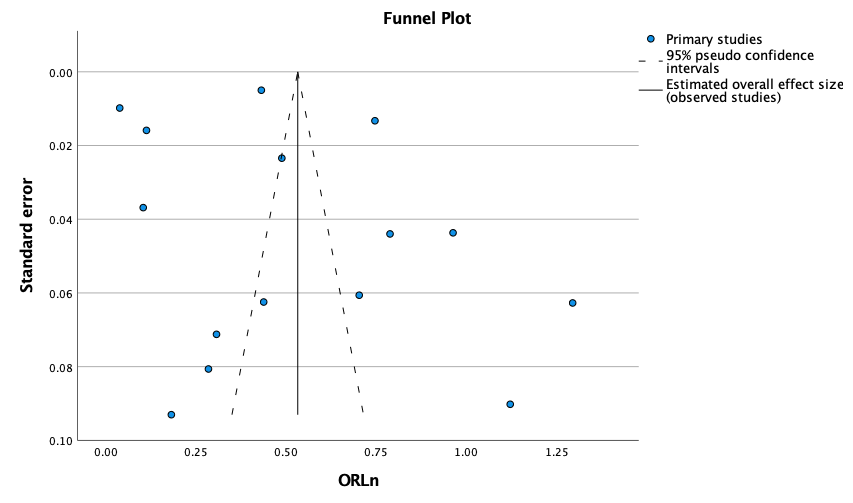


**Subgroup ethnicity**

**Black**


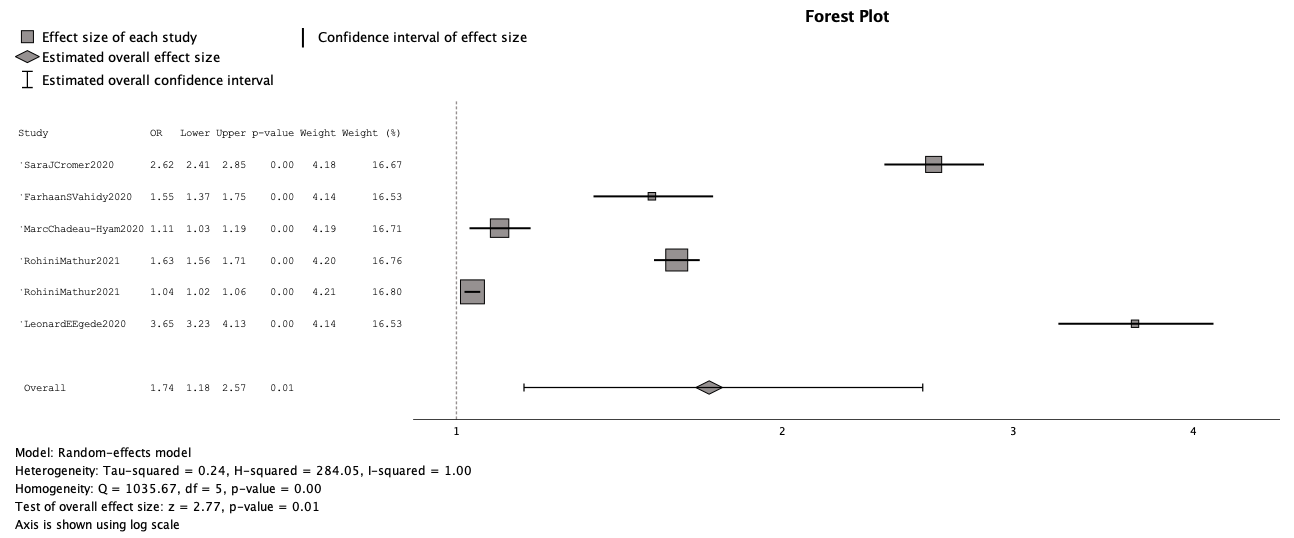


**
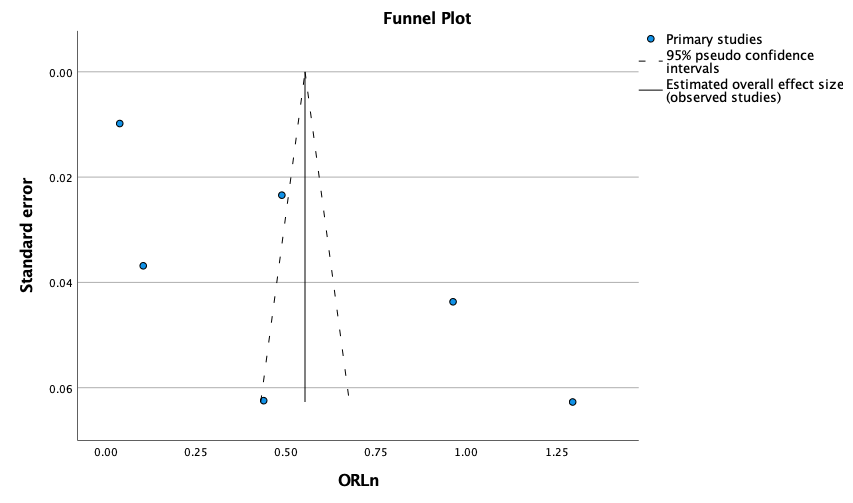
**

**Hispanic**


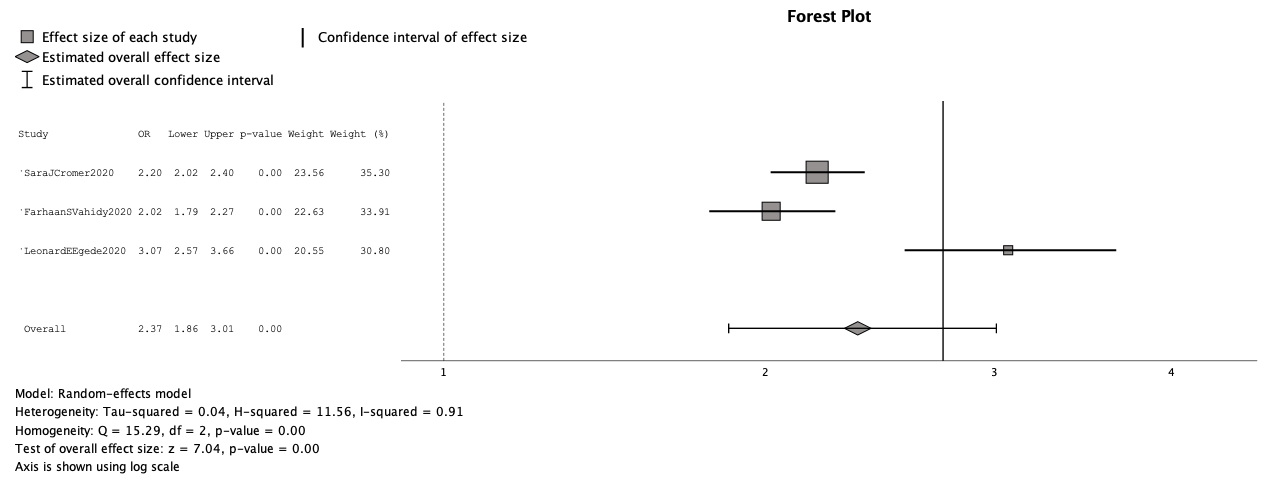


**
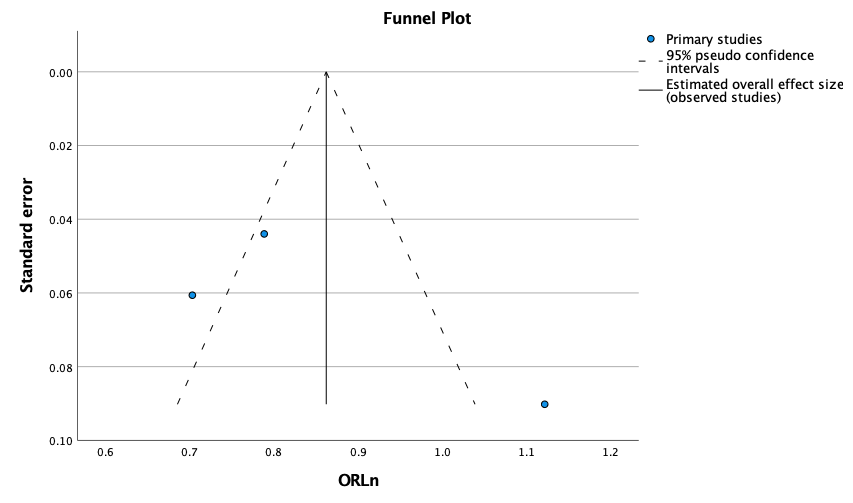
**

**Asian**


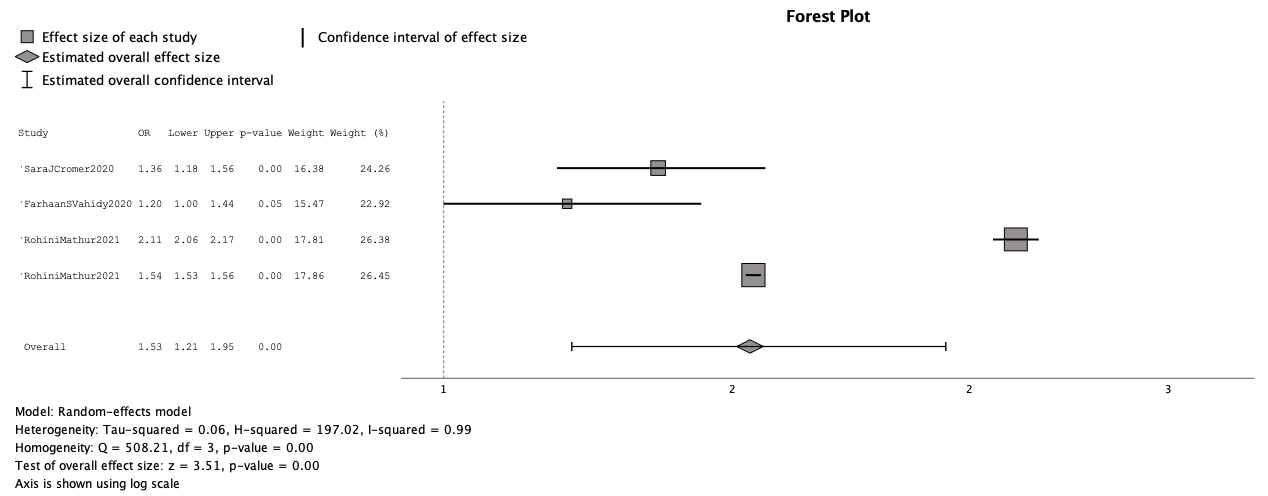


**
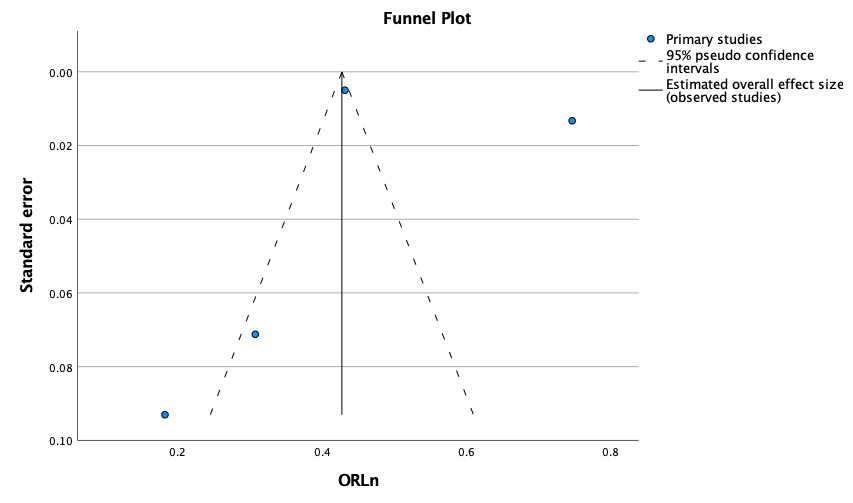
**

**Household number**


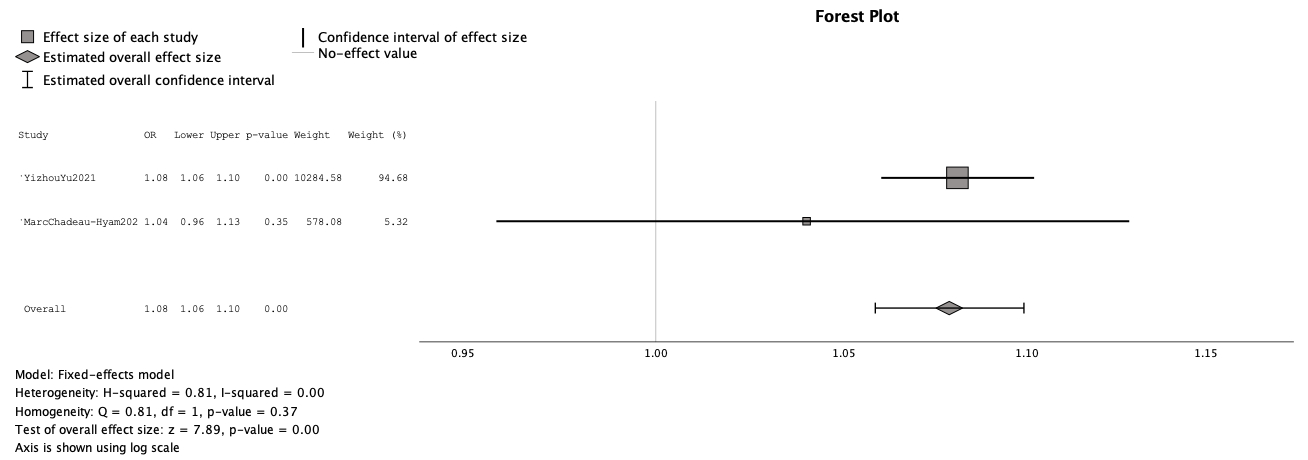


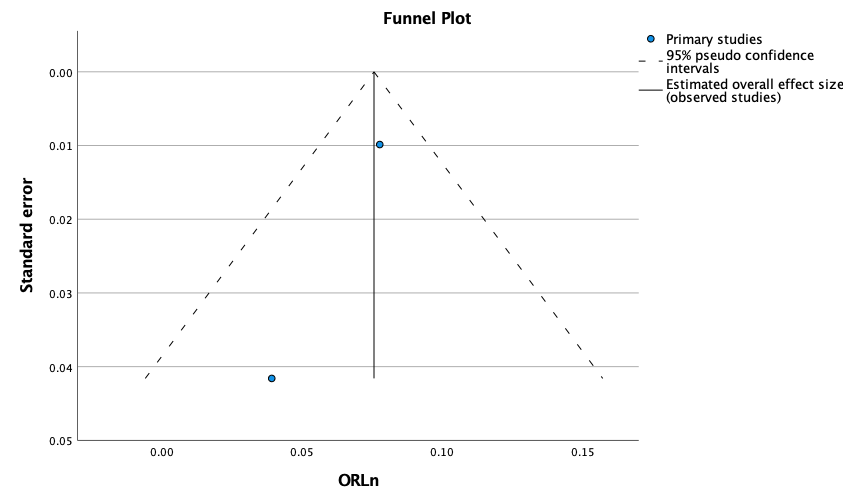


**Asthma**


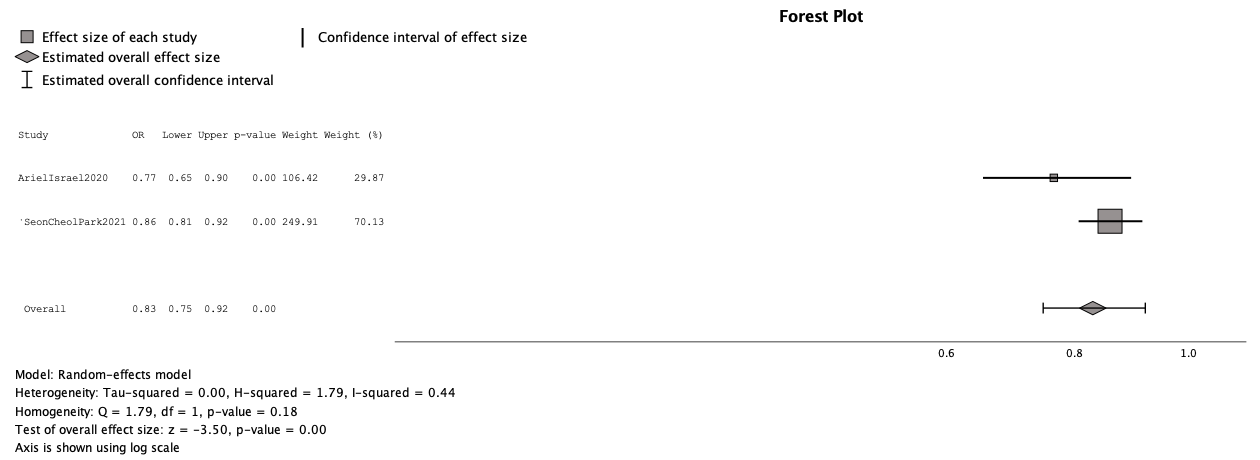


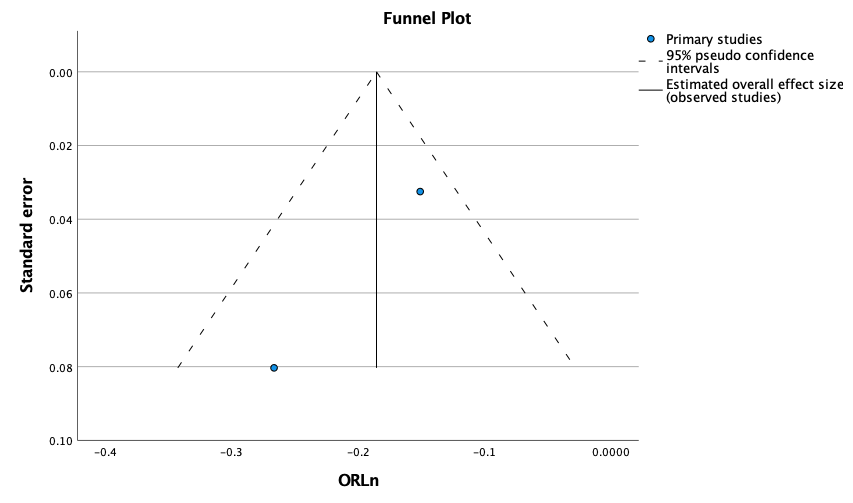


**Obesity**


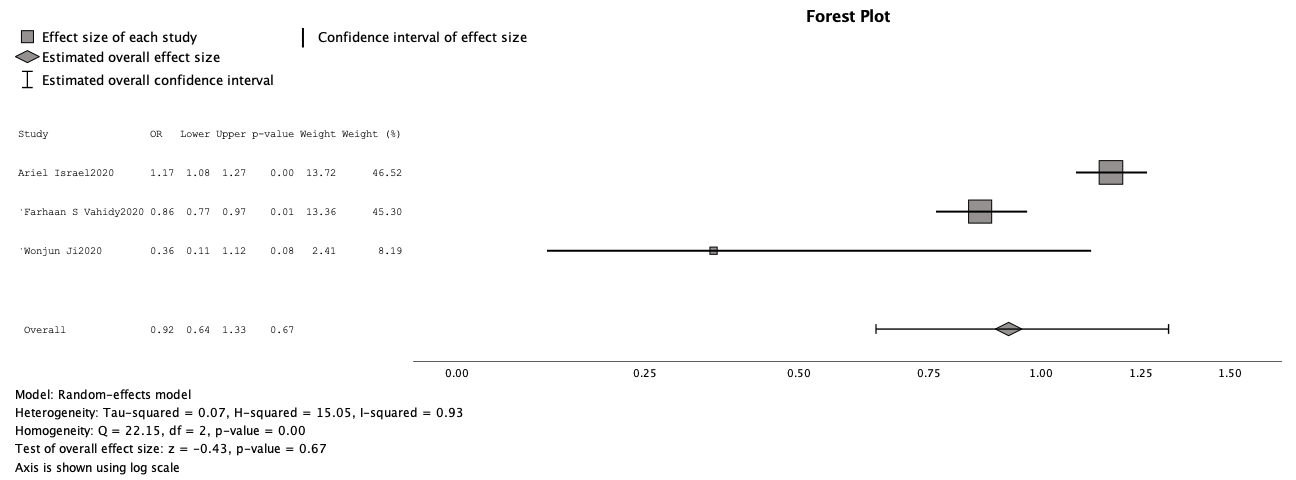


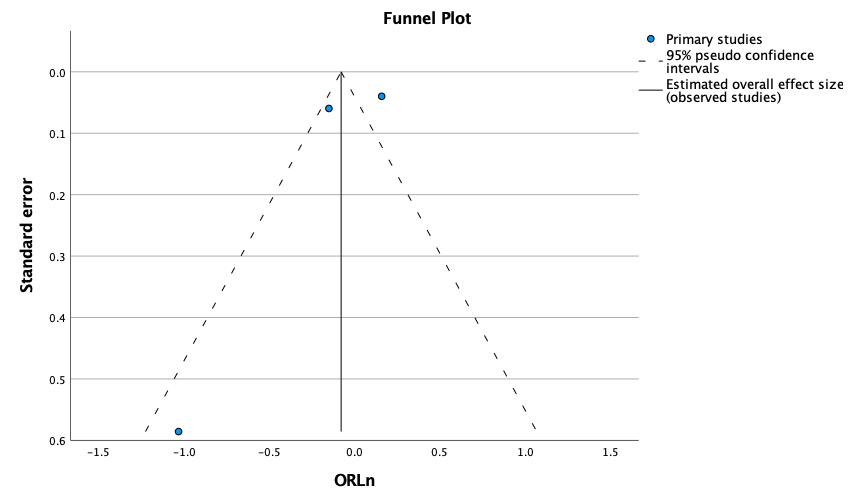


**ACE inhibitors**


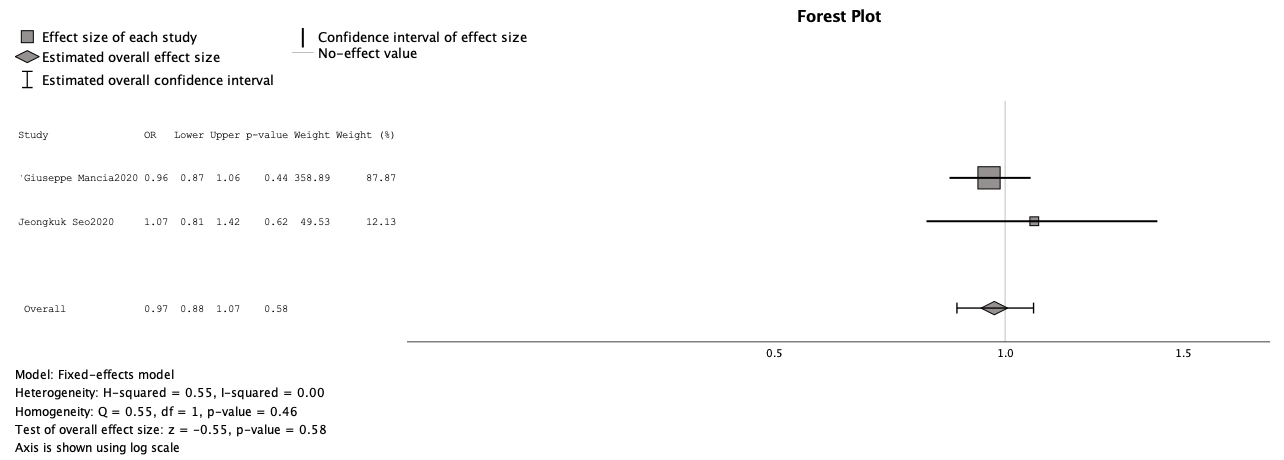


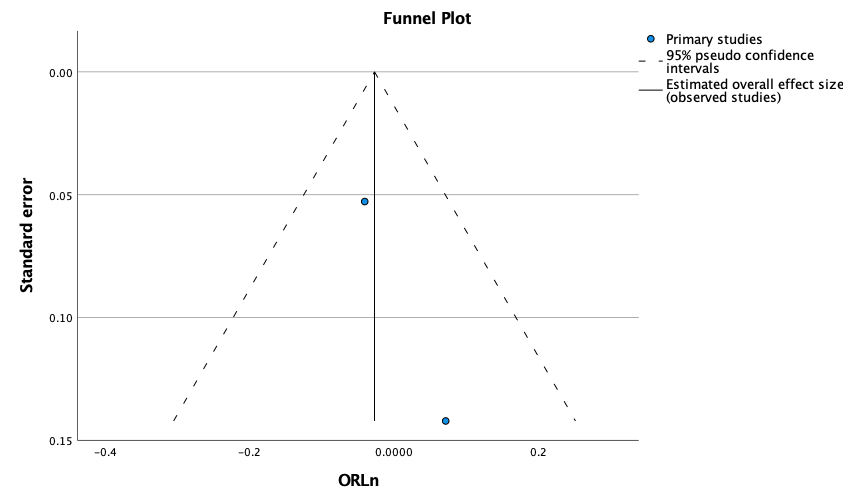


**ARBs**


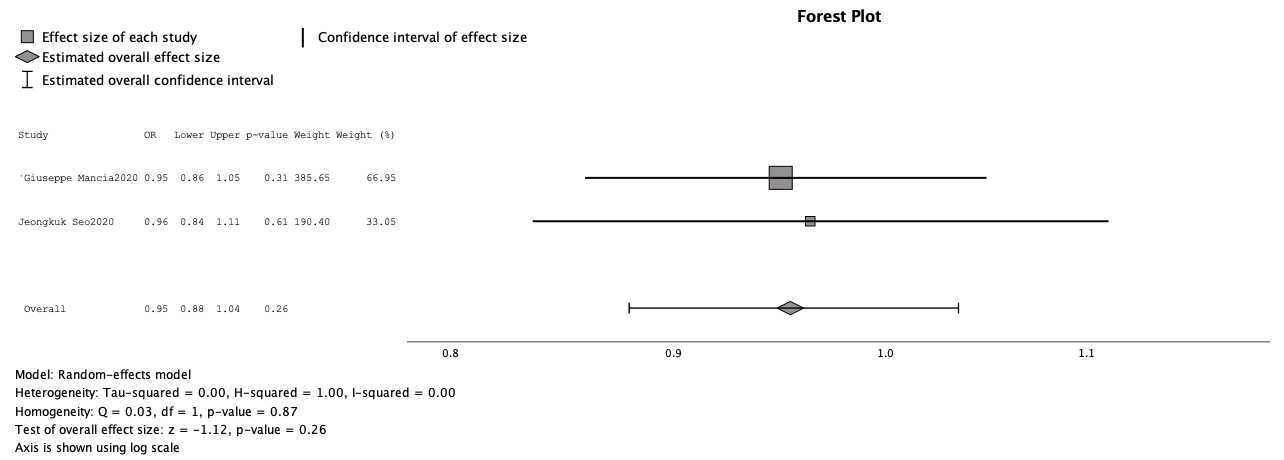


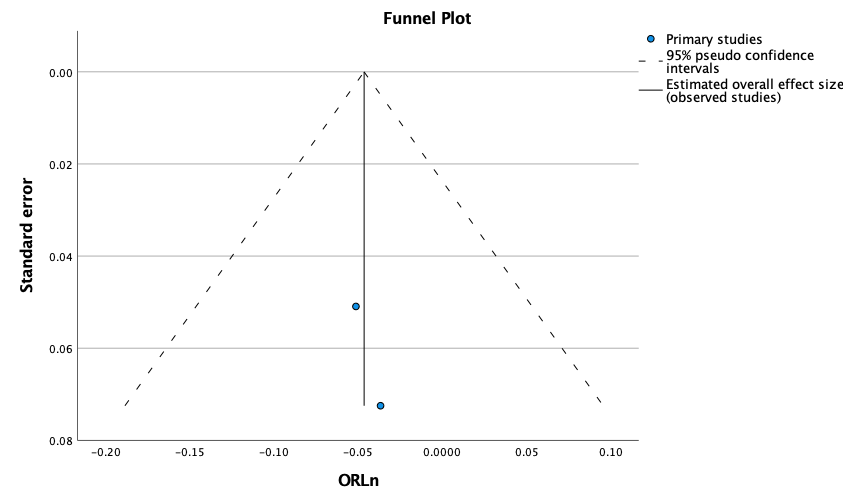


**Alzheimer**


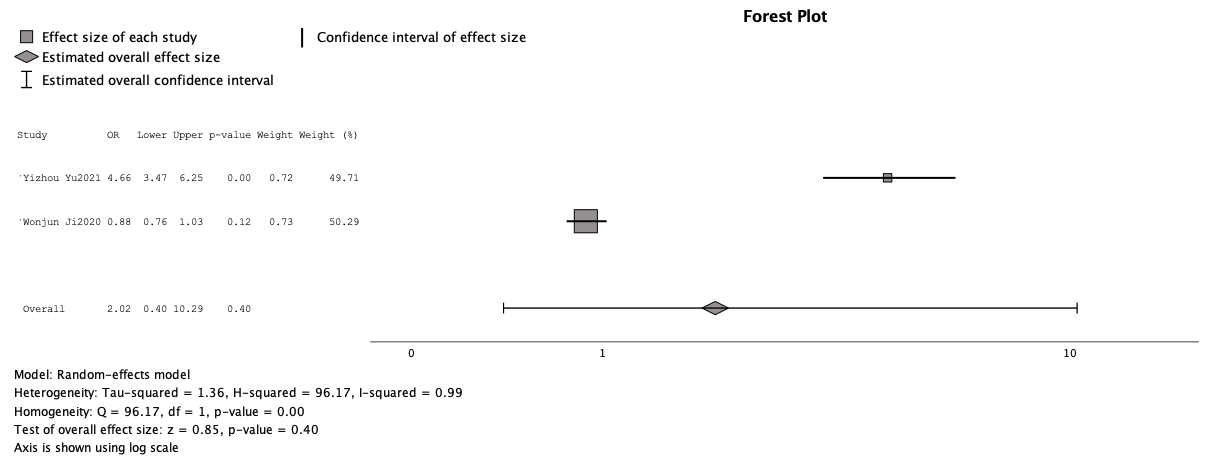


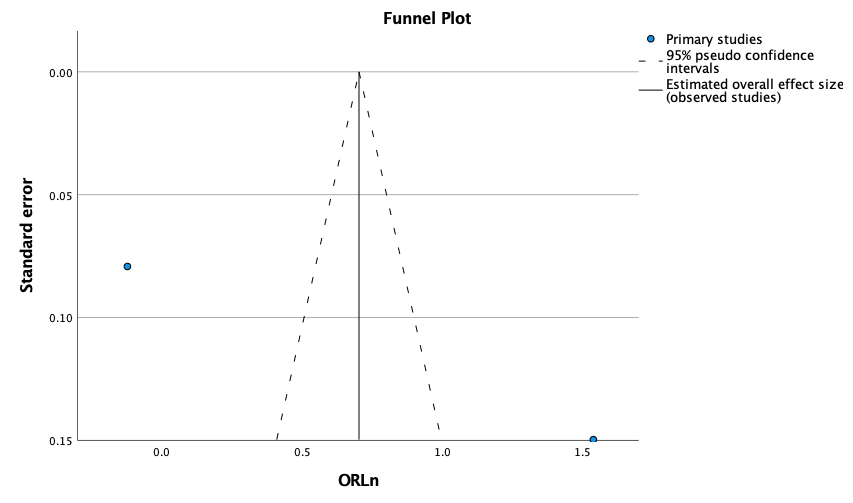


**Dementia**


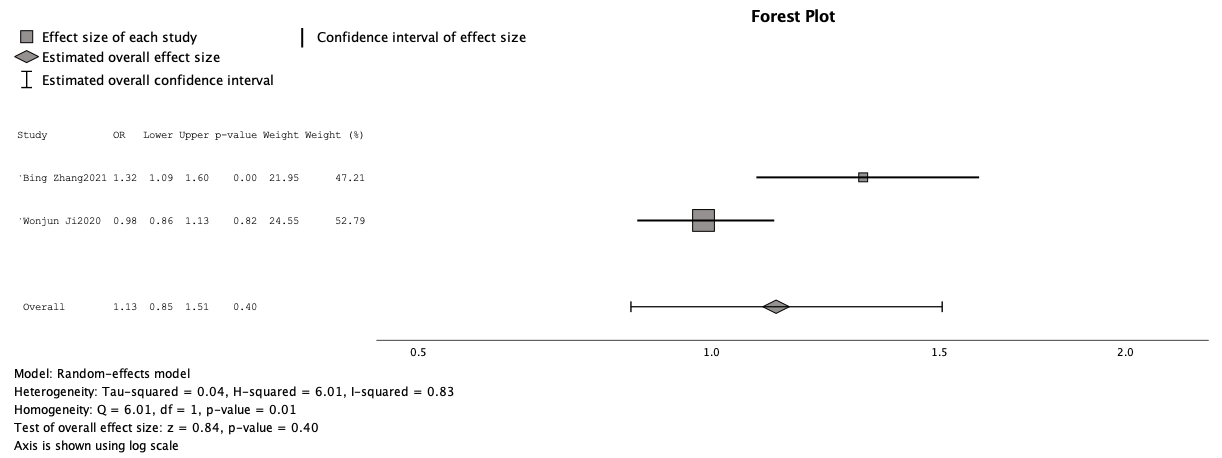


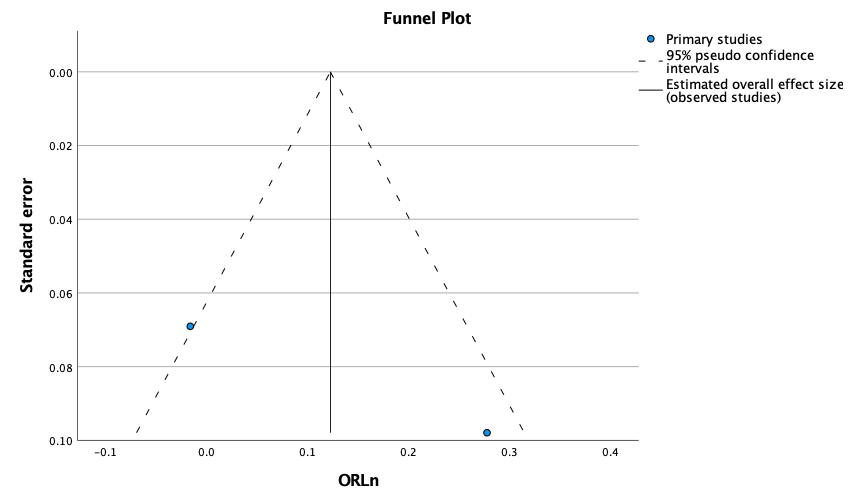


**Cardiovascular**


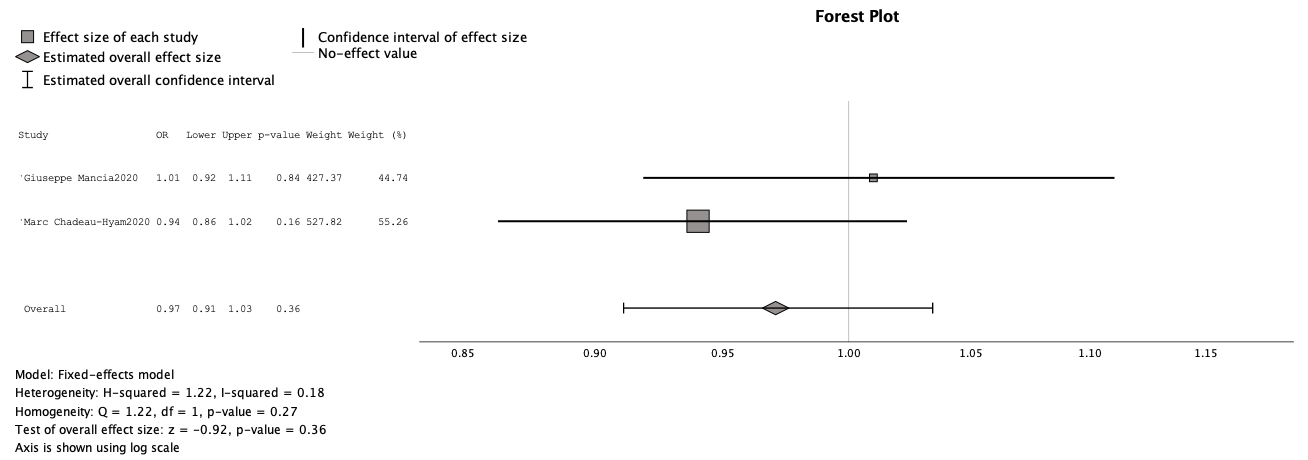


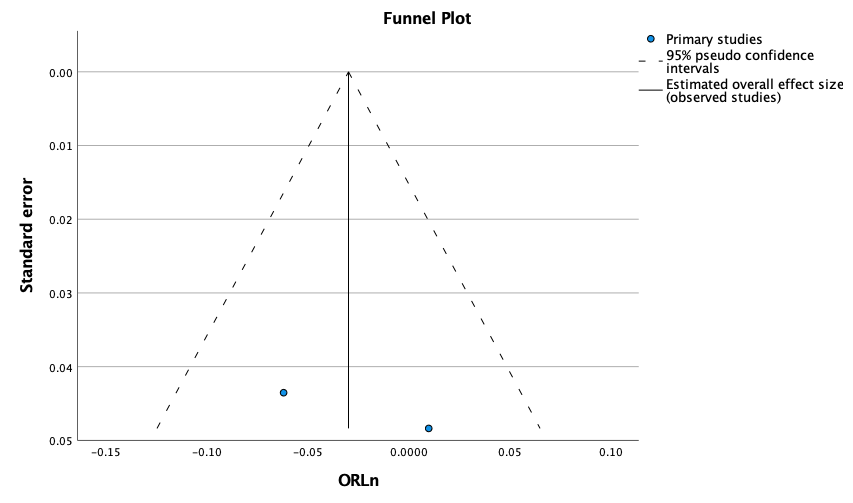


**COPD**


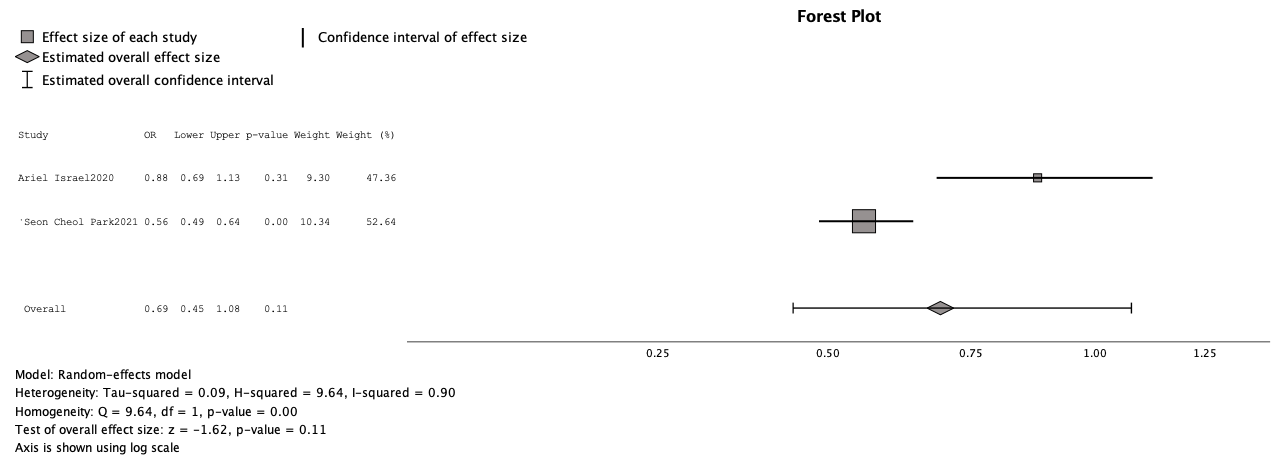


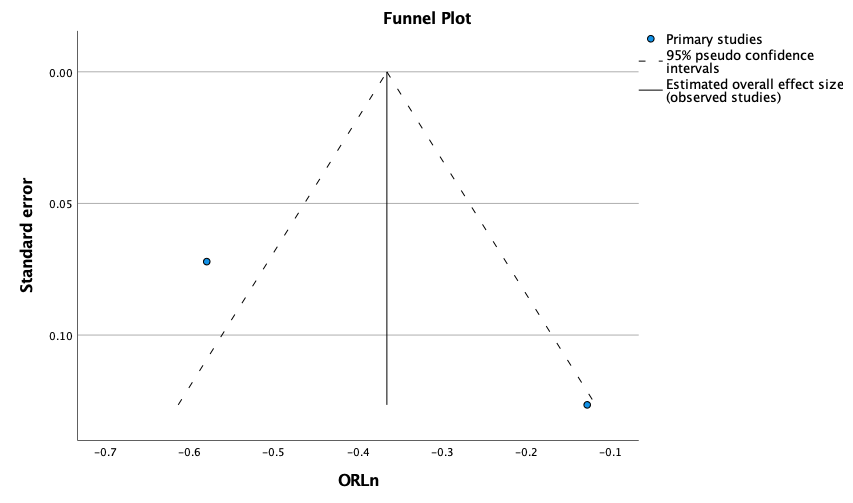


**Arrhythmia**


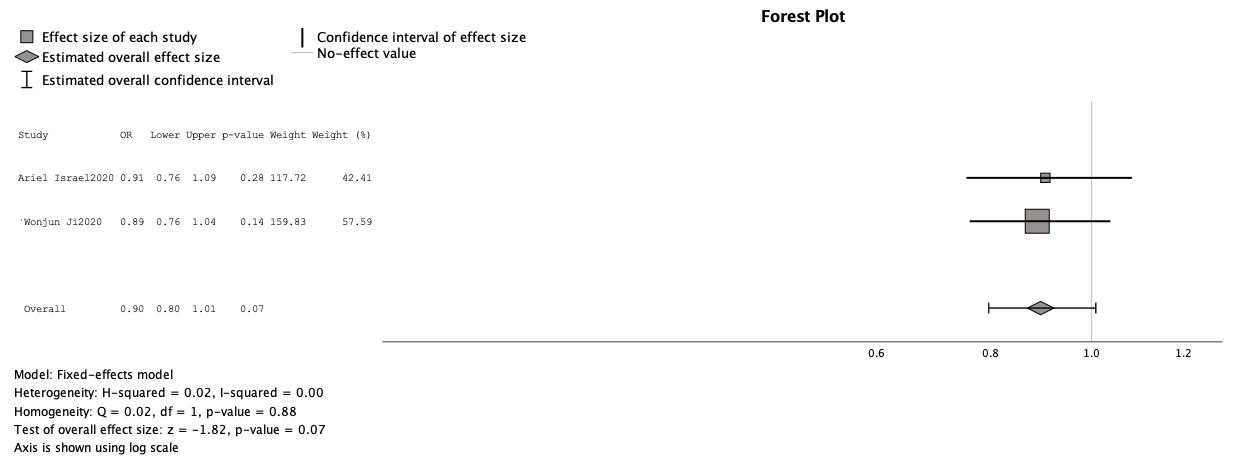


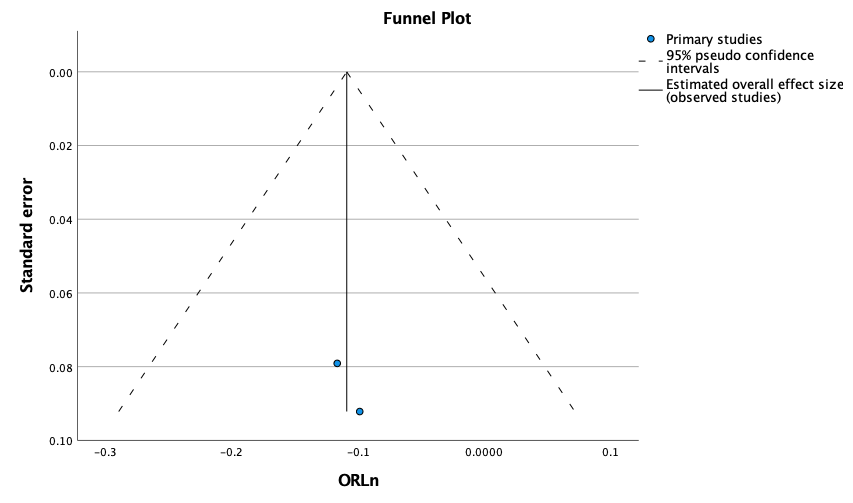


**Ischemic heart disease**


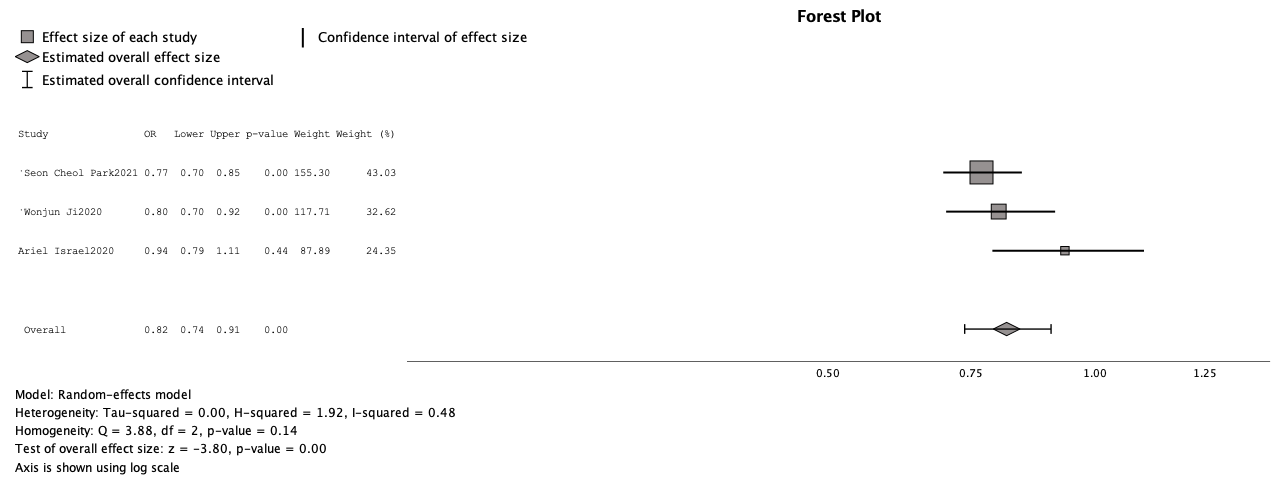


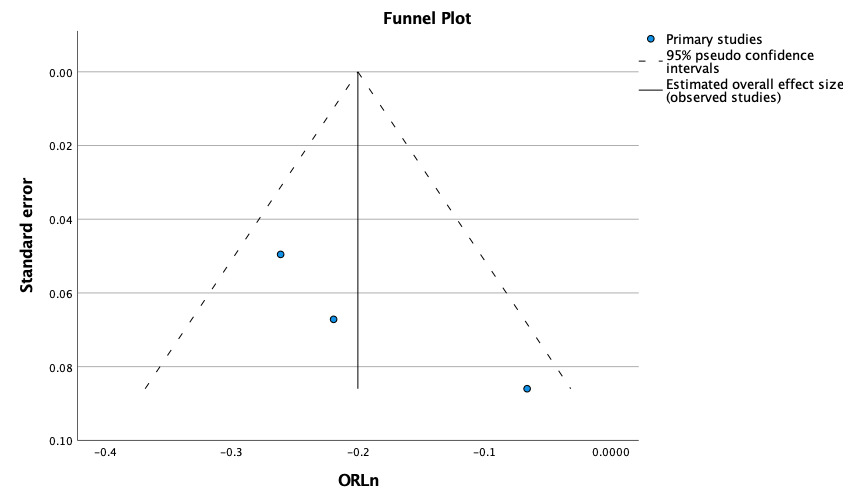


**Liver cirrhosis**


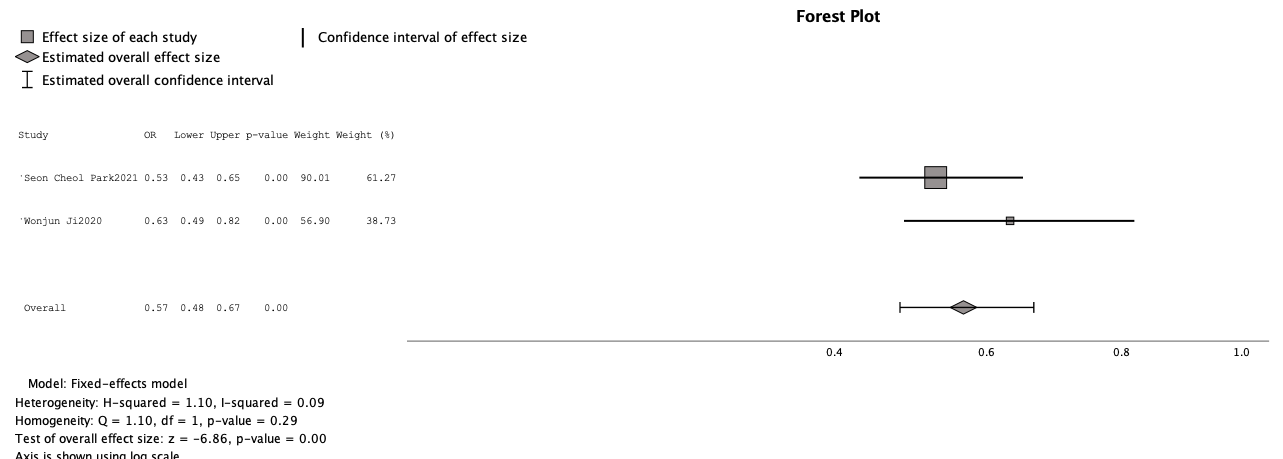


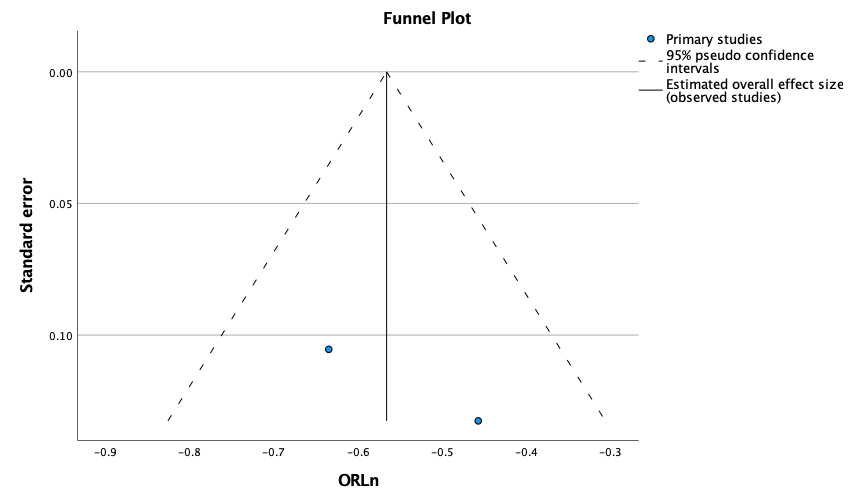


**Rheumatoid arthritis**


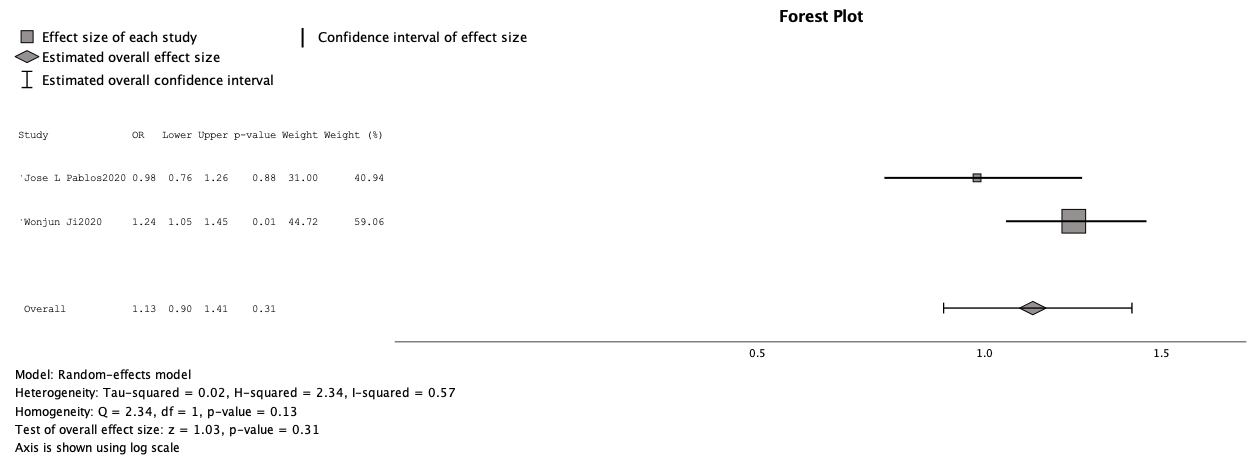


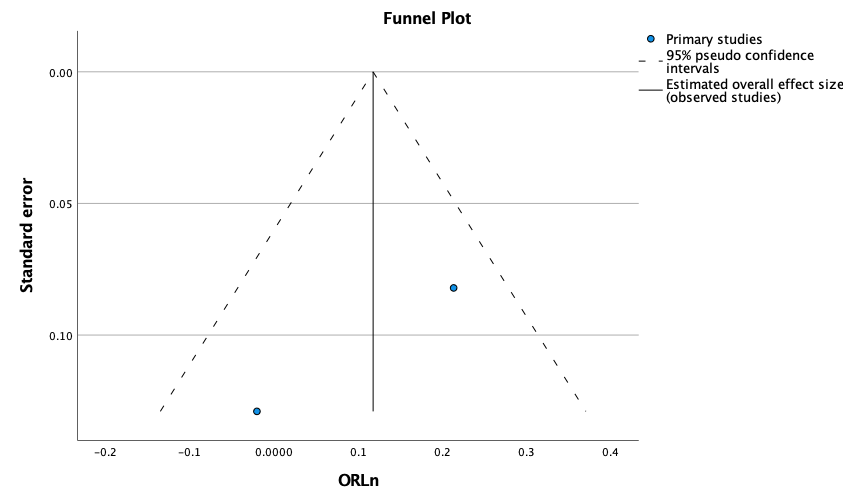


**Diabetes**


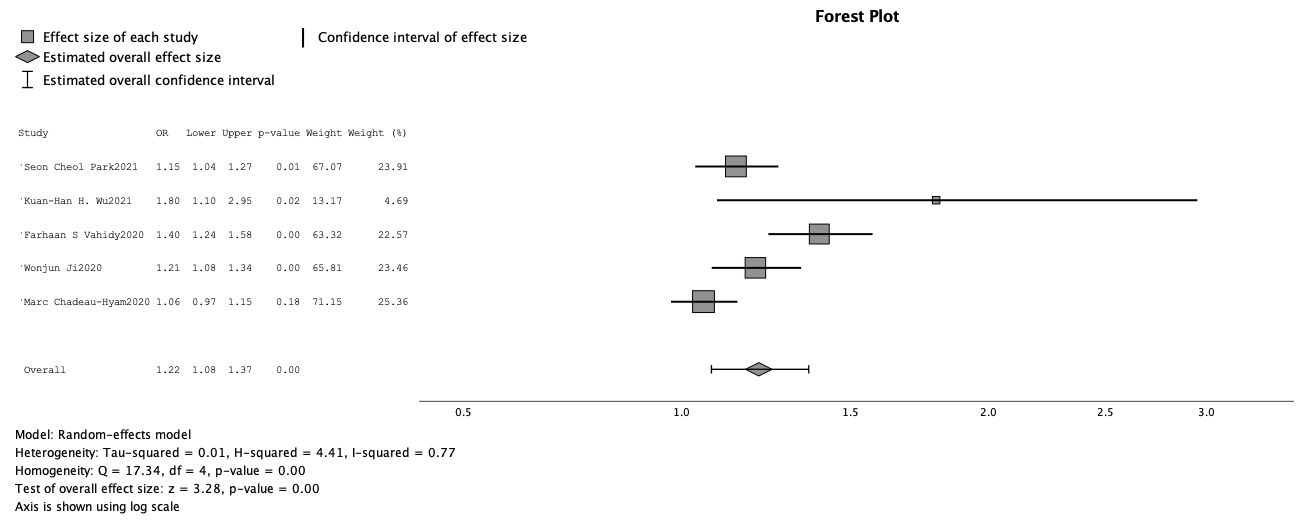


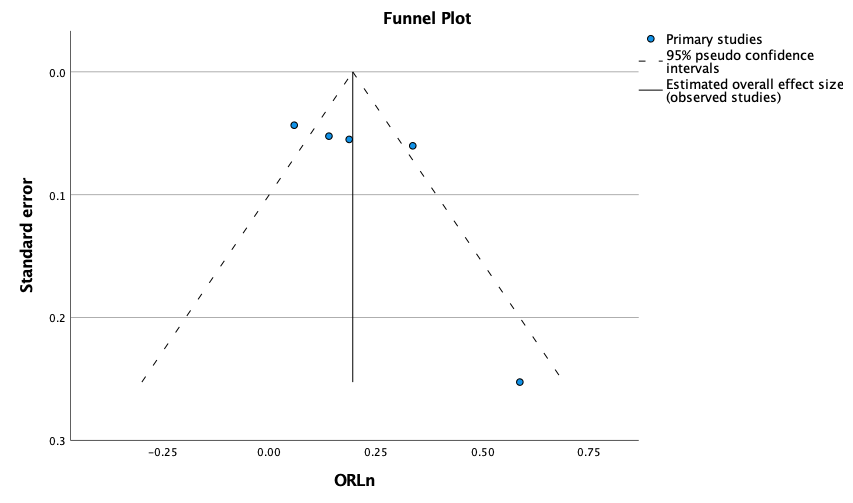


**Diabetes** (type II)


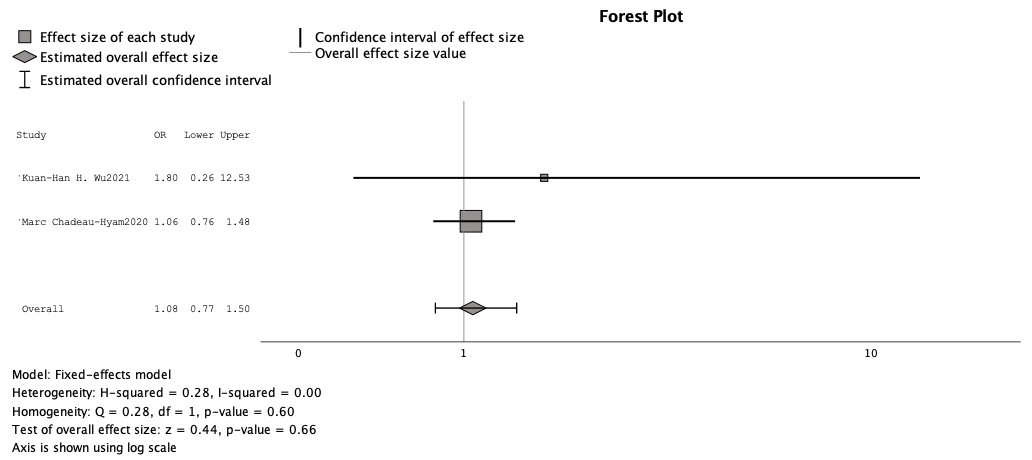


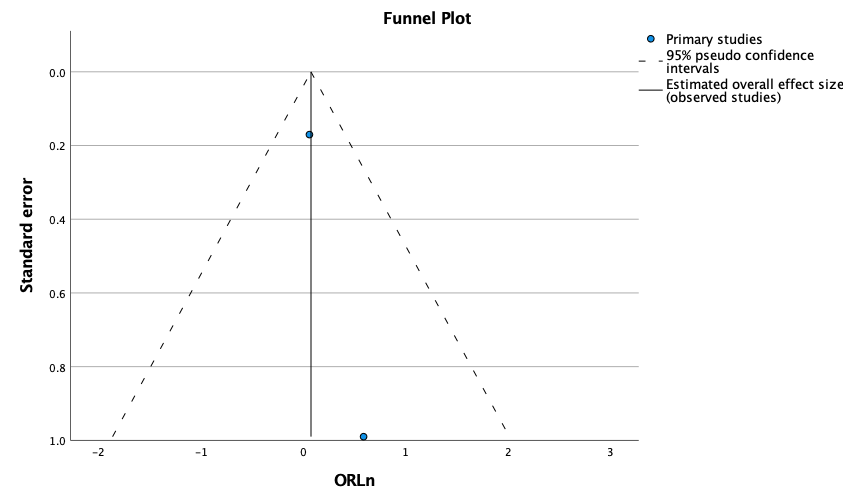


**Hypertension**


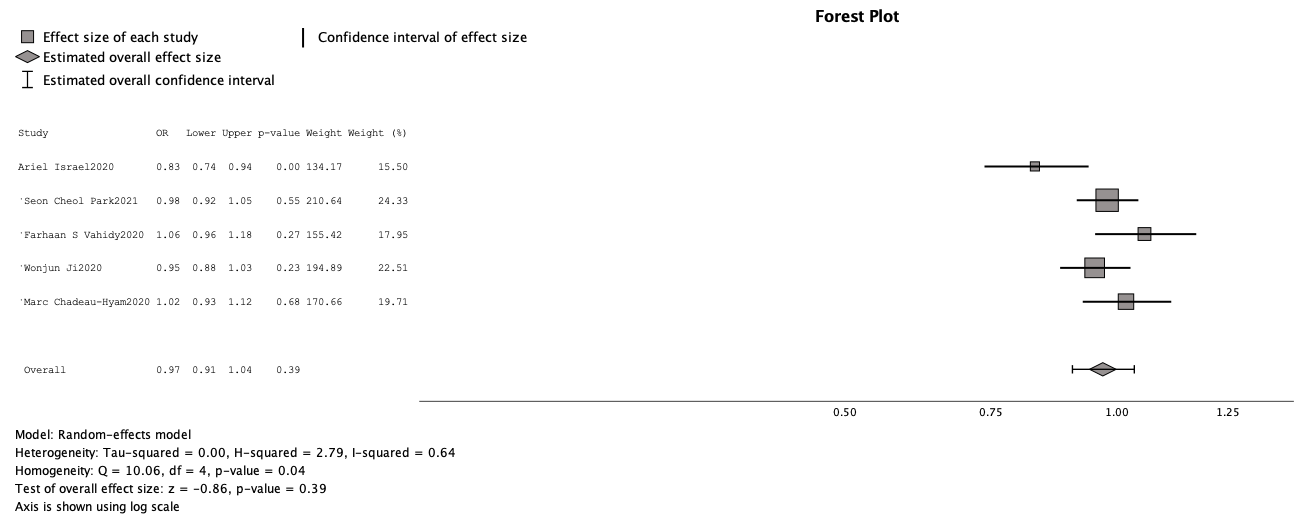


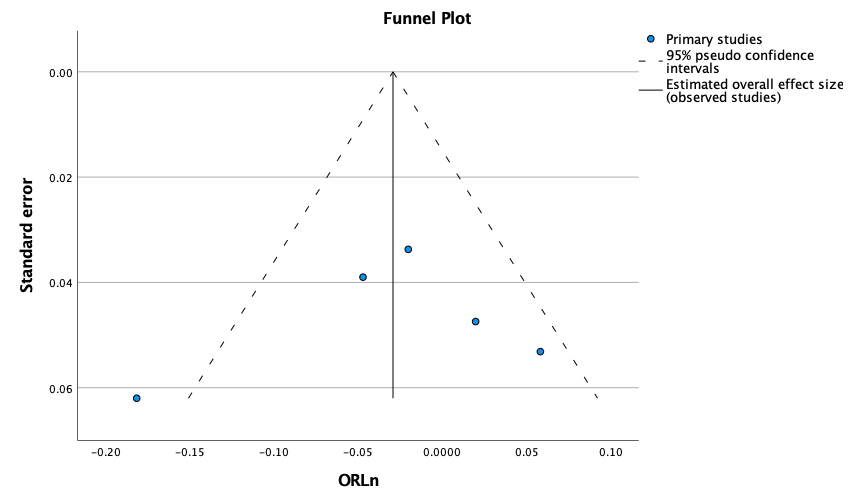


**Respiratory diseases**


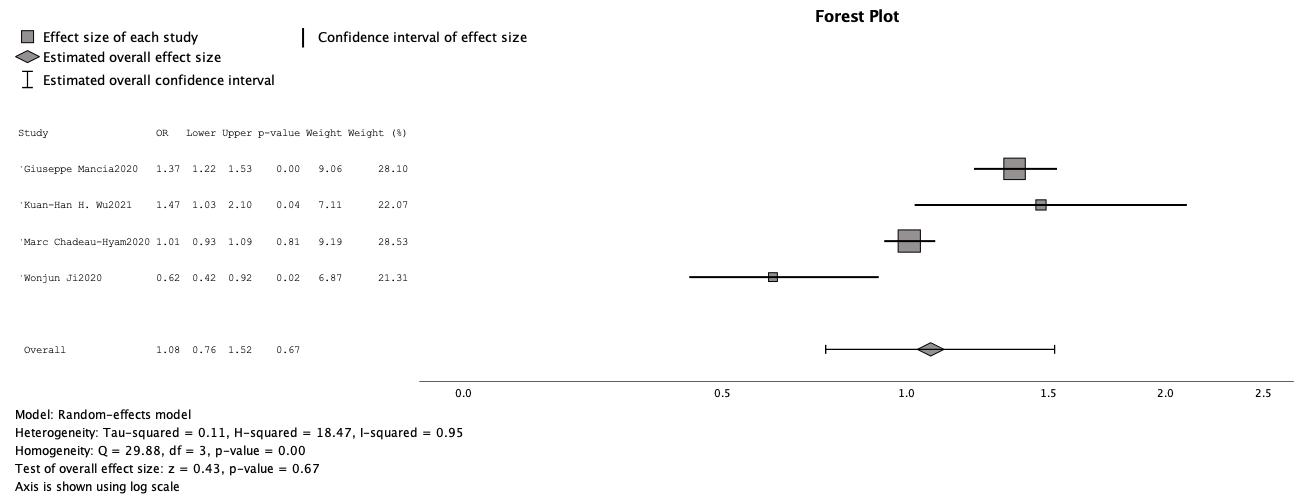


**
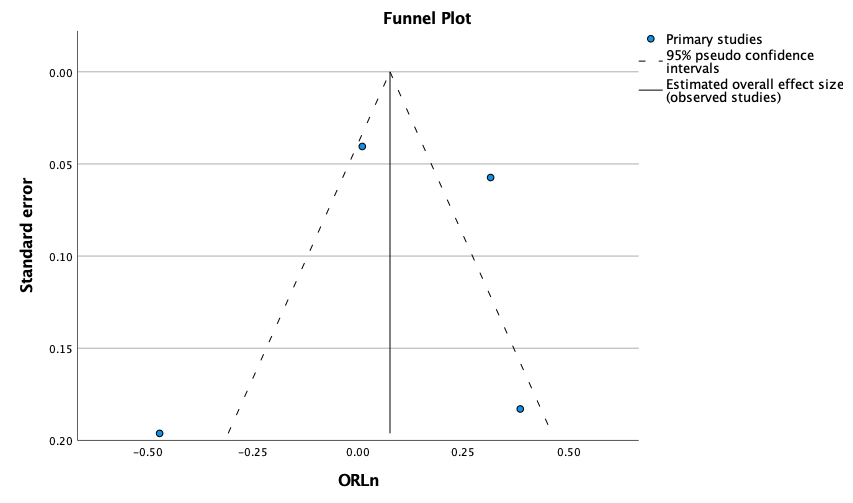
**

**Heart failure**


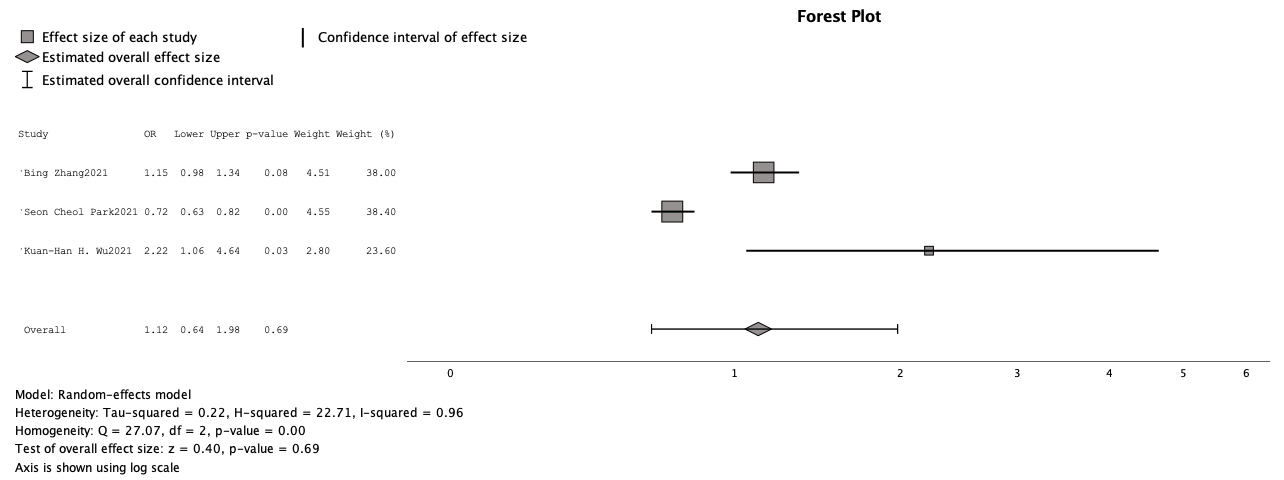


**
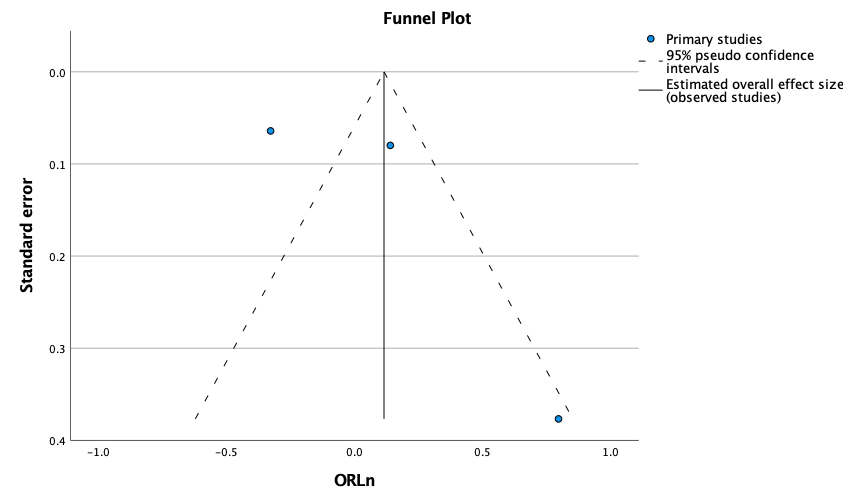
**

**Cerebrovascular diseases**


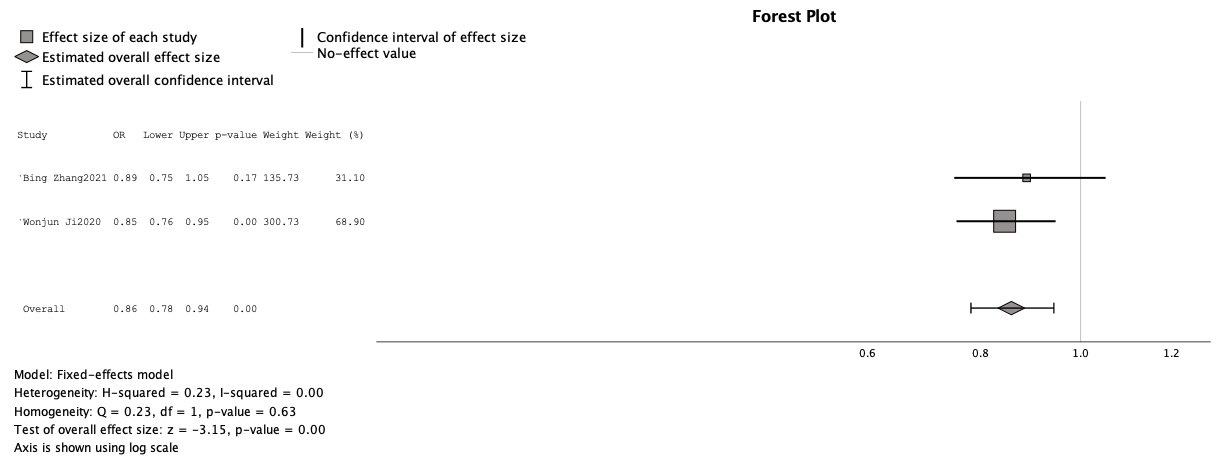


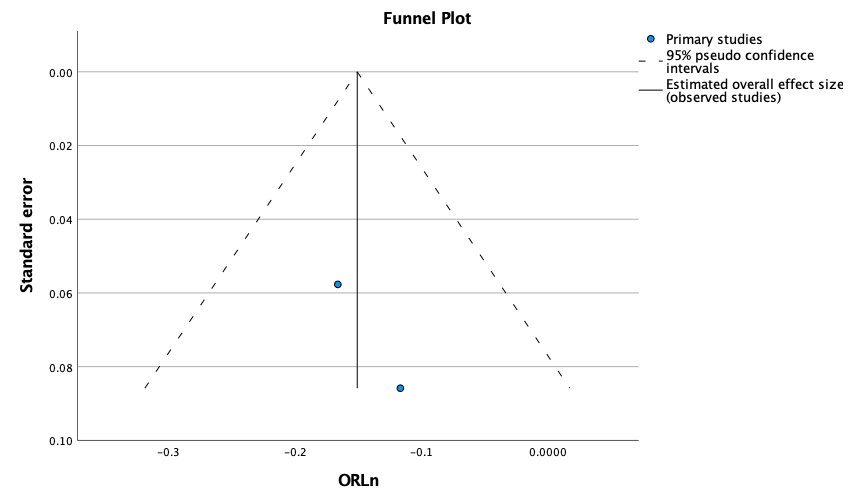


**Autoimmune diseases**


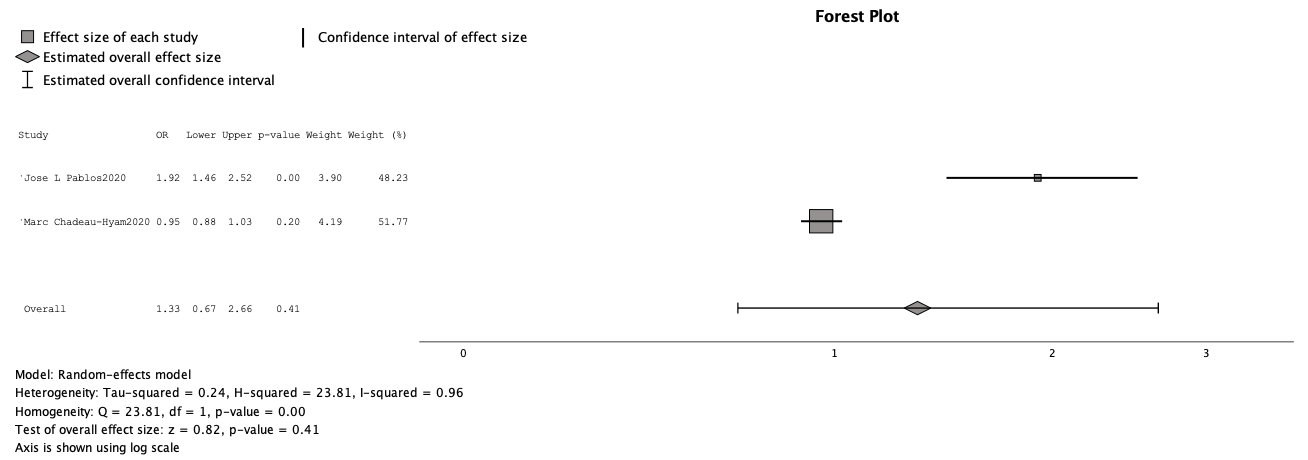


**
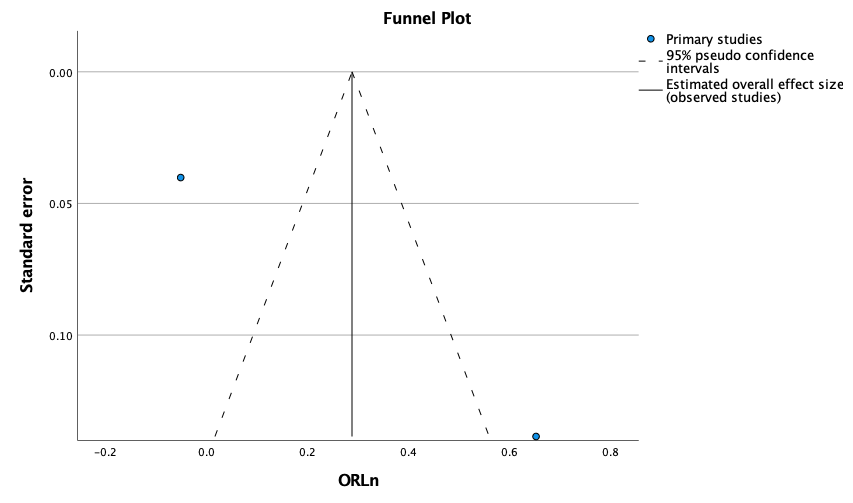
**

**Insurance**


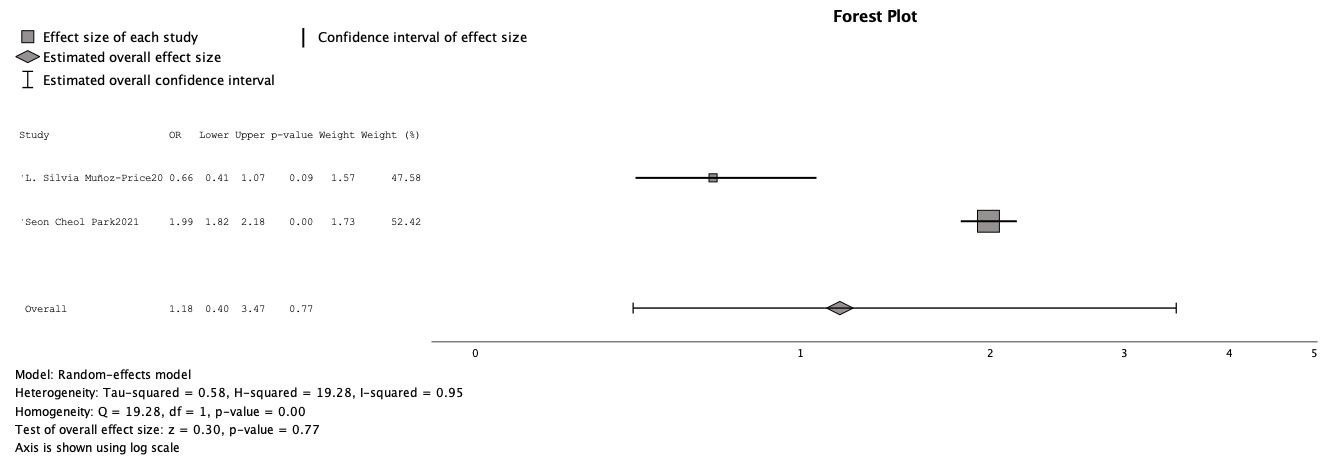


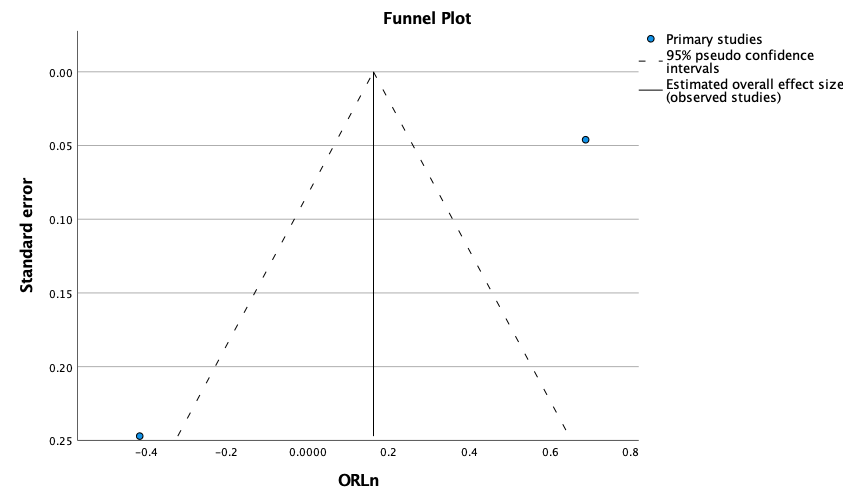


**Health worker**


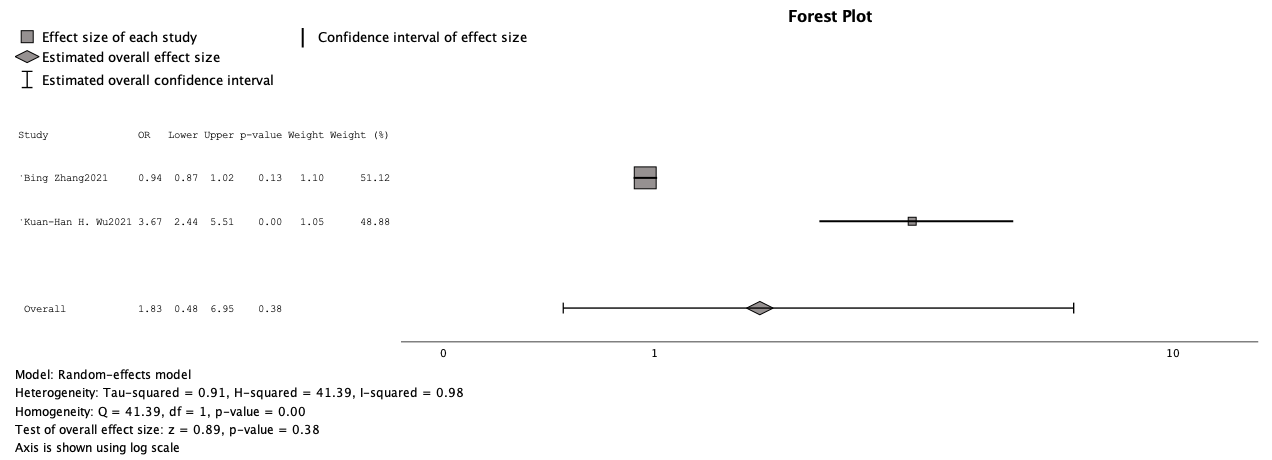


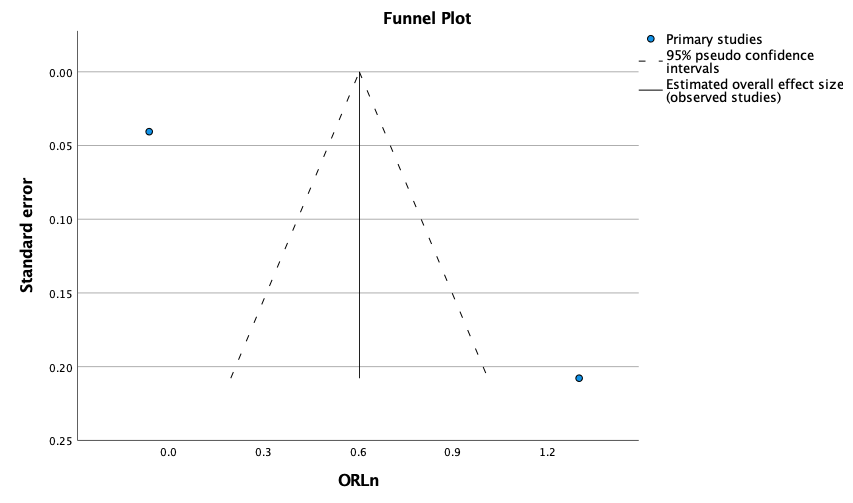


**Cancer**


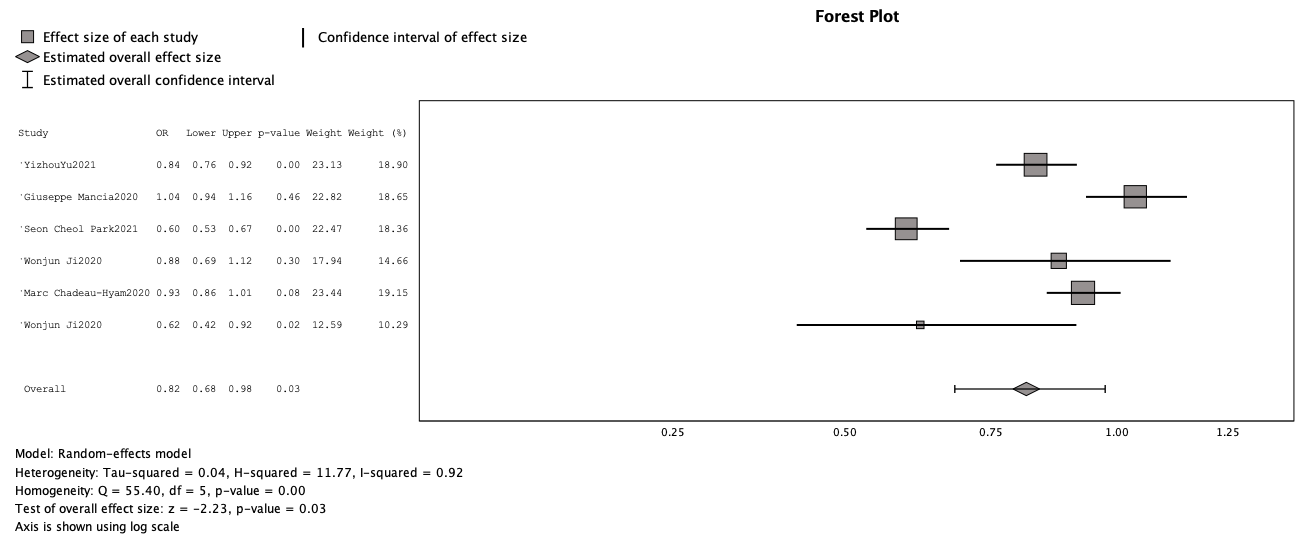


**
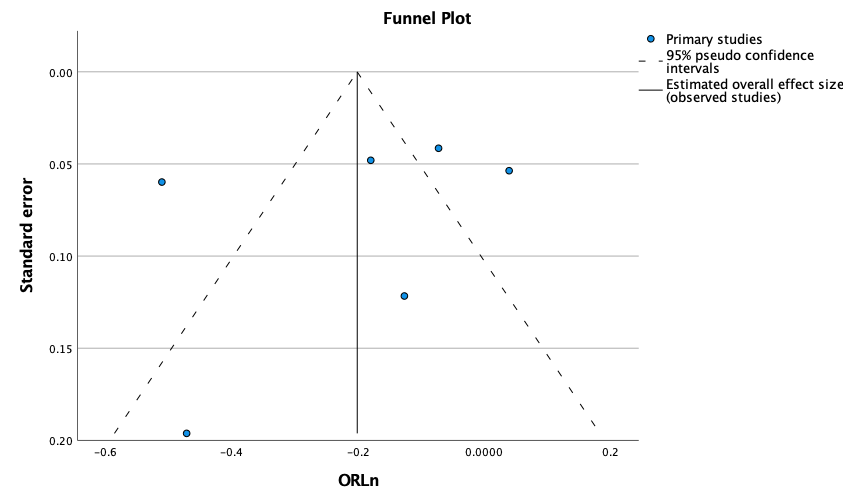
**

**Group of diseases**

**Antihypertensive drugs**


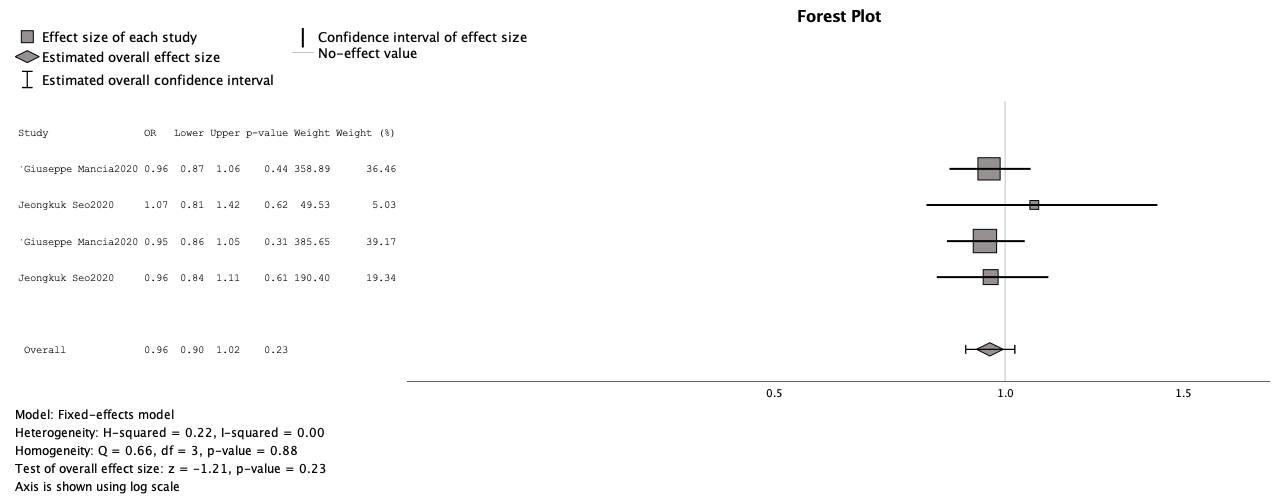


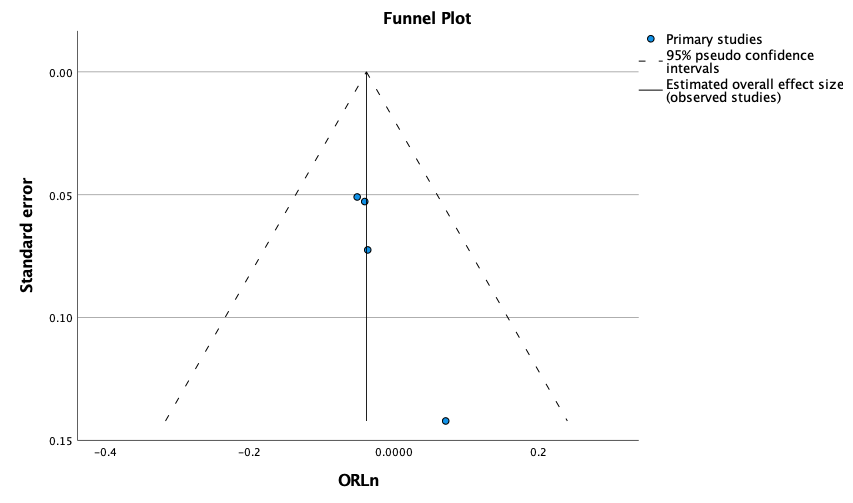


**Respiratory diseases**


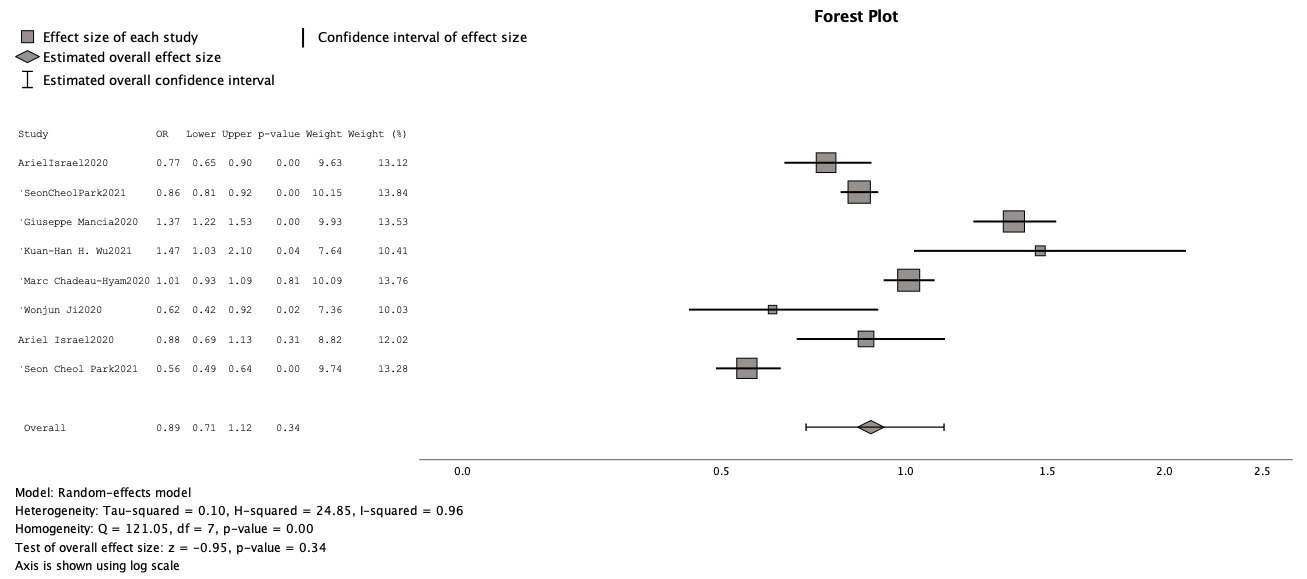


**
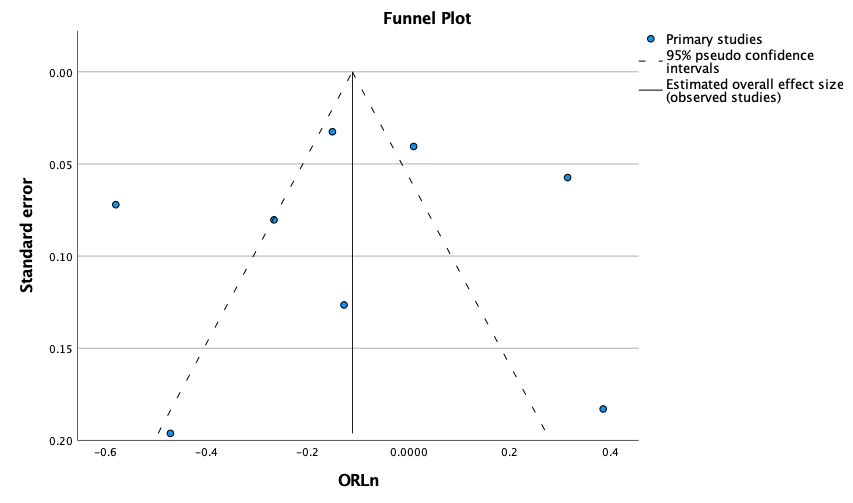
**

**Autoimmune diseases**


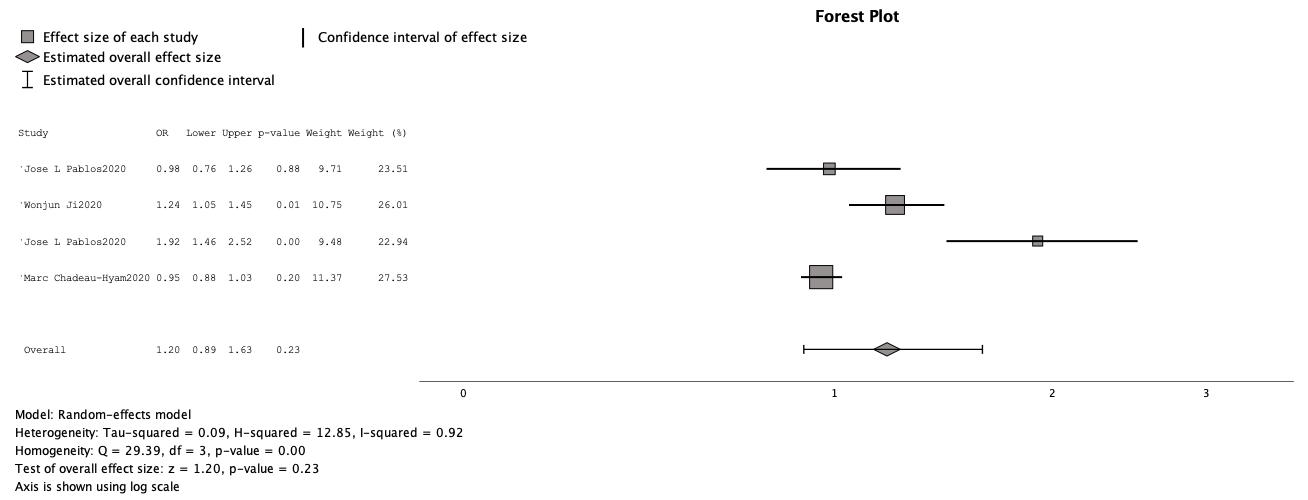


**
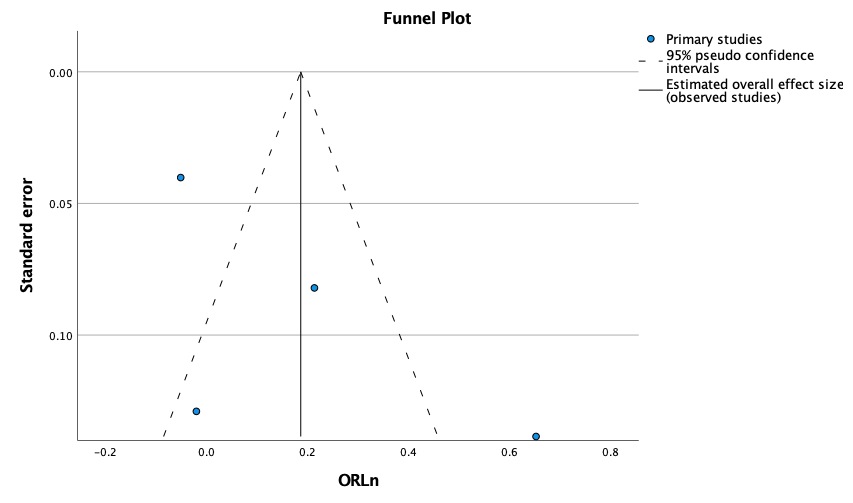
**

**Neuro diseases**


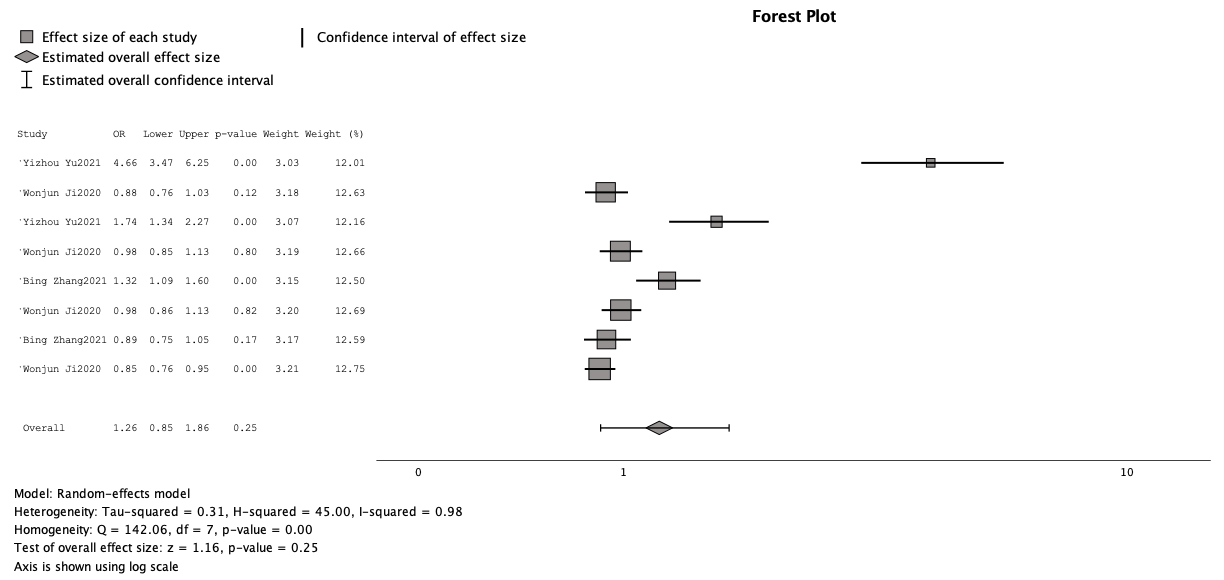


**
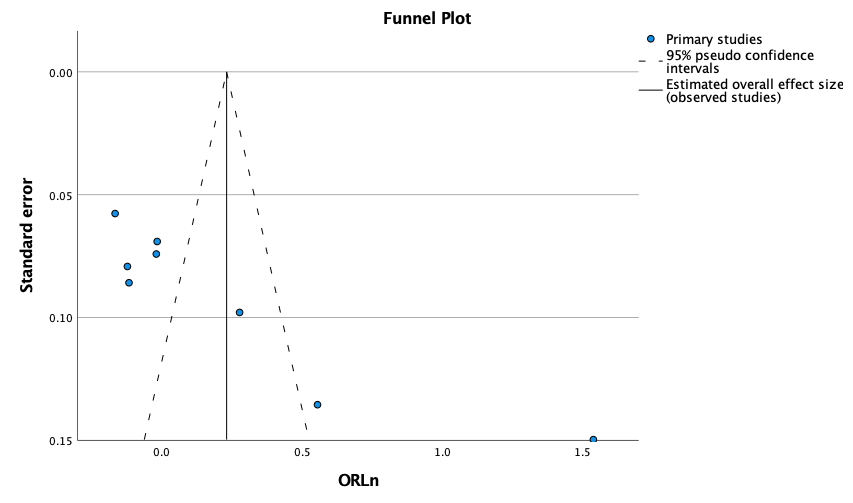
**

**Cardiovascular diseases**


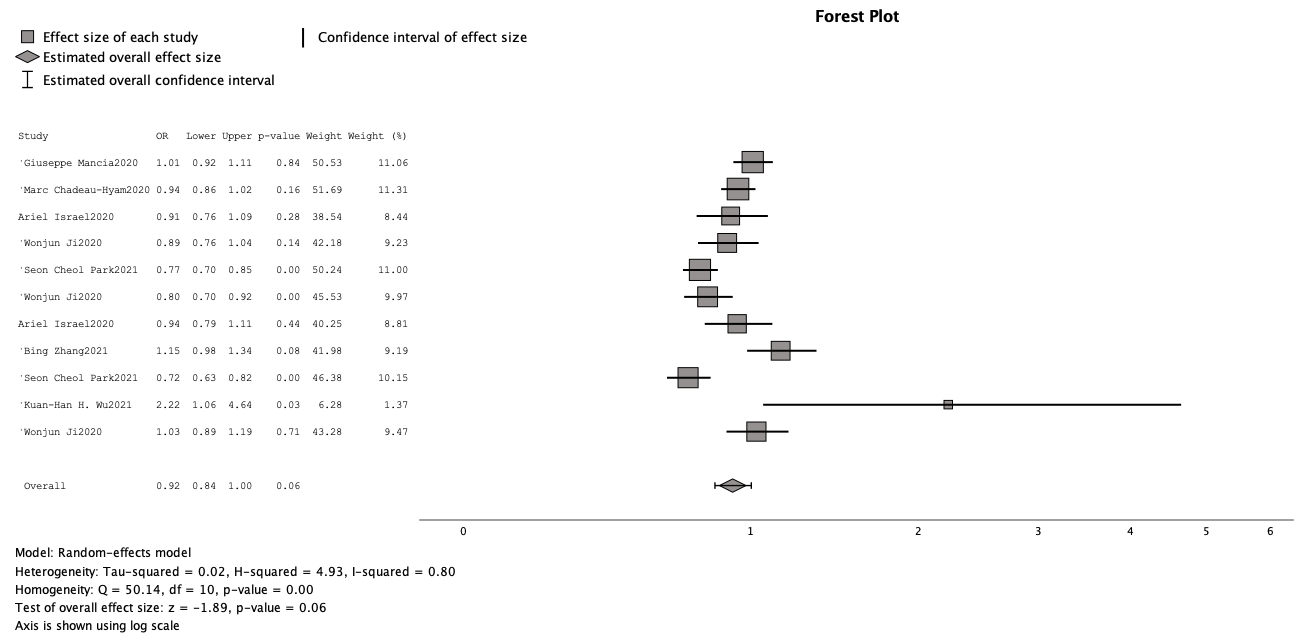


**
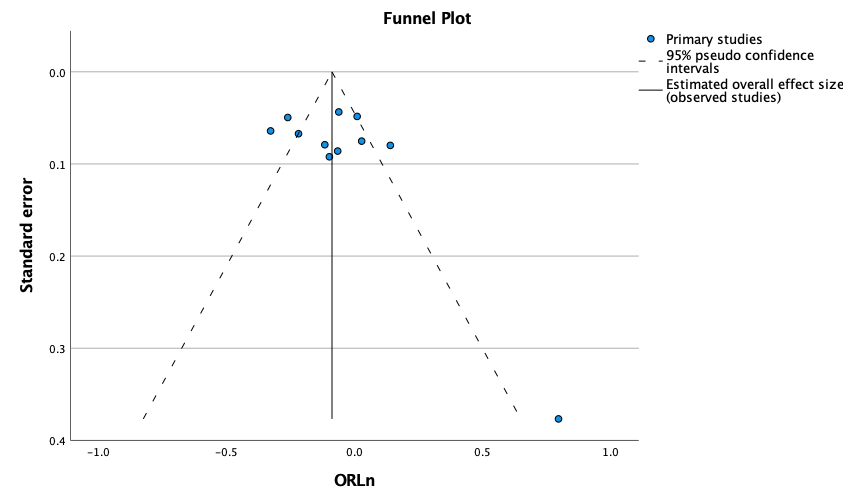
**
