# Supplementary material for: Individual risk factors associated with SARS-CoV-2 infection during Alpha variant in high-income countries: a systematic review and meta-analysis
Source: Front Public Health. 2024 Jul 30;12:1367480. doi: 10.3389/fpubh.2024.1367480 (PMC11319152; doi:10.3389/fpubh.2024.1367480)
Supplement: Supplementary file 1 [file Data_Sheet_1.ZIP › SF6_Diseases list.docx]

**Respiratory diseases**: ﻿chronic obstructive pulmonary disease[1,2], asthma[1,2], respiratory tract malignancy[3], sleep apnea[2], use of a home CPAP[2], emphysema[2], cystic Fibrosis[2], and see table[4].

**Cardiovascular diseases:** ﻿coronary artery disease[1], percutaneous coronary intervention[1], heart failure[1], and see table[4].

**Autoimmune diseases**: see table[4].

| **Cardiovascular** | | | **Respiratory** | | | **Autoimmune (1/2)** | | | **Autoimmune (2/2)** | | |
| --- | --- | --- | --- | --- | --- | --- | --- | --- | --- | --- | --- |
| 3 | ICD-10 | ICD-9 | Field 20002 | ICD-10 | ICD-9 | Field 20002 | ICD-10 | ICD-9 | Field 20002 | ICD-10 | ICD-9 |
| 1066 | I00-I02 | 390-392 | 1111 | J30-J39 | 470-478 | 1222 | D68.3 | 286.5 | 1426 | L14 | 250.93 |
| 1067 | I05-I09 | 393-398 | 1112 | J40-J47 | 480-488 | 1224 | D68.4 | 286.7 | 1435 | L40 | 255.41 |
| 1068 | I20-I25 | 410-414 | 1113 | J60-J70 | 490-496 | 1225 | D68.5 | 289.81 | 1453 | L43 | 340 |
| 1074 | I26-I28 | 415-417 | 1114 | J80-J84 | 500-508 | 1226 | D69.3 | 287.31 | 1461 | L63.9 | 357.81 |
| 1075 | I30-I52 | 420-429 | 1115 | J85-J86 | 510-519 | 1228 | D86 | 135 | 1462 | L80 | 358 |
| 1076 | I60-I69 | 430-438 | 1117 | J90-J94 |  | 1234 | D89 | 245.2 | 1463 | L90.0 | 377.3 |
| 1077 | I70-I76 | 440-445 | 1120 | J95-J99 |  | 1256 | E06.3 | 250.01 | 1464 | M05 | 447.6 |
| 1078 | I77.0-I77.5 | 447.0-447.5 | 1121 |  |  | 1260 | E10 | 250.03 | 1475 | M06 | 555 |
| 1079 | I77.7-I77.9 | 447.7-447.9 | 1122 |  |  | 1261 | E27.1 | 250.11 | 1477 | M07 | 556 |
| 1080 | I78-I79 | 448-449 | 1123 |  |  | 1313 | E27.2 | 250.13 | 1480 | M08 | 571.6 |
| 1081 | I81-I82 | 453 | 1124 |  |  | 1331 | G35 | 250.21 | 1481 | M30 | 571.42 |
| 1082 | I95-I99 |  | 1125 |  |  | 1345 | G61 | 250.23 | 1506 | M31 | 576.1 |
| 1083 |  |  | 1126 |  |  | 1371 | G70 | 250.31 | 1520 | M32 | 579.0 |
| 1086 |  |  |  |  |  | 1372 | H46 | 250.33 | 1522 | M33 | 694 |
| 1087 |  |  |  |  |  | 1376 | I77.6 | 250.41 | 1549 | M34 | 696 |
| 1088 |  |  |  |  |  | 1377 | K50 | 250.43 | 1550 | M35 | 697.0 |
| 1093 |  |  |  |  |  | 1378 | K51 | 250.51 | 1561 | M45 | 704.01 |
| 1094 |  |  |  |  |  | 1379 | K74.3 | 250.53 | 1564 | M46.0 | 709.01 |
|  |  |  |  |  |  | 1380 | K75.4 | 250.61 | 1609 | M46.1 | 701.0 |
|  |  |  |  |  |  | 1381 | K83.01 | 250.63 | 1661 | M46.2 | 714 |
|  |  |  |  |  |  | 1382 | K90.0 | 250.71 | 1667 | M46.4 | 446 |
|  |  |  |  |  |  | 1383 | L10 | 250.73 |  | M46.8 | 710 |
|  |  |  |  |  |  | 1384 | L11 | 250.81 |  | M46.9 | 720.0 |
|  |  |  |  |  |  | 1397 | L12 | 250.83 |  | N08 | 720.8 |
|  |  |  |  |  |  | 1428 | L13 | 250.91 |  | D51.0 | 281.0 |

[1] Mancia G, Rea F, Ludergnani M, Apolone G, Corrao G. Renin–Angiotensin–Aldosterone System Blockers and the Risk of Covid-19. N Engl J Med 2020;382:2431–40. https://doi.org/10.1056/NEJMoa2006923.

[2] Wu K-HH, Hornsby WE, Klunder B, Krause A, Driscoll A, Kulka J, et al. Exposure and risk factors for COVID-19 and the impact of staying home on Michigan residents. PLoS One 2021;16:e0246447. https://doi.org/10.1371/journal.pone.0246447.

[3] Ji W, Huh K, Kang M, Hong J, Bae GH, Lee R, et al. Effect of Underlying Comorbidities on the Infection and Severity of COVID-19 in Korea: a Nationwide Case-Control Study. J Korean Med Sci 2020;35:e237. https://doi.org/10.3346/jkms.2020.35.e237.

[4] Chadeau-Hyam M, Bodinier B, Elliott J, Whitaker MD, Tzoulaki I, Vermeulen R, et al. Risk factors for positive and negative COVID-19 tests: A cautious and in-depth analysis of UK biobank data. Int J Epidemiol 2020;49:1454–67. https://doi.org/10.1093/ije/dyaa134.
